# A chromosome-level genome assembly of a model conifer plant, the Japanese cedar, *Cryptomeria japonica* D. Don

**DOI:** 10.1186/s12864-024-10929-4

**Published:** 2024-11-05

**Authors:** Takeshi Fujino, Katsushi Yamaguchi, Toshiyuki T. Yokoyama, Toshiya Hamanaka, Yoritaka Harazono, Hiroaki Kamada, Wataru Kobayashi, Tokuko Ujino-Ihara, Kentaro Uchiyama, Asako Matsumoto, Ayako Izuno, Yoshihiko Tsumura, Atsushi Toyoda, Shuji Shigenobu, Yoshinari Moriguchi, Saneyoshi Ueno, Masahiro Kasahara

**Affiliations:** 1 Graduate School of Frontier Sciences, The University of Tokyo, Kashiwa, 277-8561 Japan; 2 Trans-Scale Biology Center, National Institute for Basic Biology, Okazaki, 444-8585 Japan; 3 Department of Forest Molecular Genetics and Biotechnology, Forestry and Forest Products Research Institute, Tsukuba, 305-8687 Japan; 4 Faculty of Life and Environmental Sciences, University of Tsukuba, Tsukuba, 305-8572 Japan; 5 Comparative Genomics Laboratory, National Institute of Genetics, Mishima, 411-8540 Japan; 6 Faculty of Agriculture, Niigata University, Niigata, 950-2181 Japan

**Keywords:** *Cryptomeria japonica*, Sugi, Genome assembly, HiFi sequencing, Hi-C scaffolding, Gene annotation, Repetitive elements, Model conifer

## Abstract

**Background:**

The Japanese cedar (*Cryptomeria japonica* D. Don) is one of the most important Japanese forest trees, occupying approximately 44% of artificial forests and planted in East Asia, the Azores Archipelago, and certain islands in the Indian Ocean. Although the huge genome of the species (ca. 9 Gbp) with abundant repeat elements may have represented an obstacle for genetic analysis, this species is easily propagated by cutting, flowered by gibberellic acid, transformed by Agrobacterium, and edited by CRISPR/Cas9. These characteristics of *C. japonica* recommend it as a model conifer species for which reference genome sequences are necessary.

**Results:**

Herein, we report the first chromosome-level assembly of *C. japonica* (2n = 22) using third-generation selfed progeny (estimated homozygosity rate = 0.96). Young leaf tissue was used to extract high molecular weight DNA (> 50 kb) for HiFi PacBio long-read sequencing and to construct an Hi-C/Omni-C library for Illumina short-read sequencing. The 29× and 26× genome coverage of HiFi and Illumina reads, respectively, for *de novo* assembly yielded 2,651 contigs (9.1 Gbp, N50 contig size 12.0 Mbp). Hi-C analysis mapped 97% of the nucleotides on 11 chromosomes. The assembly was verified through comparison with a consensus linkage map comprising 7,781 markers. BUSCO analysis identified ∼ 91% conserved genes.

**Conclusions:**

Annotations of genes and comparisons of repeat elements with other Cupressaceae and Pinaceae species provide a fundamental resource for conifer research.

**Supplementary Information:**

The online version contains supplementary material available at 10.1186/s12864-024-10929-4.

## Background

The Cupressaceae, comprising the most widely distributed coniferous trees that inhabit Northern and Southern Hemispheres [1] and all continents, except Antarctica [1], comprises the highest number of genera among conifer families [2]. Due to its high resistance to decay [3–5], the wood of Cupressaceae is highly valued. In Japan, the most widely used representative of the family is the Japanese cedar (*Cryptomeria japonica* D. Don), which is a close relative of *Sequoiadendron* and *Taxodium* species in North America [6]. Although the artificial planting of *C. japonica* has been conducted since 1,400 [7], the timber of Japanese cedar has been utilized from more than 3,000 years [8], and it is therefore recommended as timber for shipbuilding due to its durability, initially described in 1896 in Japan [9]. Because of its rapid growth, adaptability to numerous environmental conditions and regeneration by cutting, grafting, and tissue culture [8, 10, 11], the species maintains a rich diversity of local varieties [8, 12, 13]. These characteristics, combined with the ease of artificial crossing, facilitated through promoting flowering by gibberellic acid [14], make it an important and intriguing resource for genetic studies. Furthermore, due to its high economic value, the species is one of the most important forestry trees in Japan as well as in subtropical China [15], Portugal (Azores Archipelago) [16], and the Reunion [17].

Although the fossil record of *Cryptomeria* is established in East Asia and Europe [18], the species now exhibits endemic distribution of the *C. japonica* var. *japonica*, var. *radicans*, and var. *sinensis*. *C. japonica* var. *japonica* and var. *radicans* exclusively localize to Japan and occupy a sparsely distributed natural range from the northernmost part of Japan’s main island, Honshu, to Yakushima an island south of Kyushu [19]. *C. japonica* var. *sinensis* is distributed in China, although the natural distribution in Fujian (Nanping), Jiangxi (Lushan mountain), and Zhejiang (Tianmu Mountain) provinces is debated [20, 21]. The Japanese government’s forest tree improvement program commenced in 1954 [7, 22], and *C. japonica* was extensively planted for timber production in the 1960s to support the country’s economic growth. Currently, the species occupies nearly 4.5 million hectares, accounting for 44% of all artificial forests in Japan [23]. Unfortunately, the proliferation of artificial forests of *C. japonica* led to widespread prevalence of Japanese cedar pollinosis, with a documented prevalence of 38.8%, representing major social issues [24]. There is therefore a pressing need to reduce the amount of *C. japonica* pollen dispersed by renewing them with male-sterile (pollen-free) trees of *C. japonica*. Indeed, sequence-based markers for male sterility that were established through this line of research [25], allow for easier identification of wild trees (for breeding) with a male-sterile allele; and the estimated frequency of trees with male-sterile alleles in breeding materials is approximately 1% [26, 27]. Thus, developing genetic markers for marker-assisted selection will be likely and significantly facilitated with chromosome-level genome assembly.

The genome assembly of conifers is difficult because of their large genomes (up to 37 Gbp) [28, 29] and an abundance of repetitive sequences compared with those of animals such as humans [30–34]. Thus, the cost of determining the genome sequence of a conifer was previously quite high. Furthermore, creating genetic maps for these species is time-consuming due to the long generation time of tree species, leading to limited application of genetic methods (e.g., genetic markers, transgenesis, and gene editing) to conifers. Breeding efforts via molecular biological techniques based on genetic maps and genome sequences are limited to only a few species. Therefore, there is a pressing need for the construction of a chromosome-level genome assembly of a conifer species to confer efficient molecular-genetic breeding. The advent of accurate long-read sequencing methods such as PacBio HiFi reads or the Oxford Nanopore kit v14 significantly decreases the cost of analyzing large genomes [33, 35, 36], leading us to conclude that it was time to establish a high-accuracy, high-contiguity model conifer genome at the chromosome scale with comprehensive gene annotation. Such a chromosome-level assembly will likely facilitate the rapid systematic identification of the relationship between diverse traits and genes (or mutations) using molecular-genetics techniques. This will enable breeding elite trees with diverse favorable quantitative and qualitative traits, including rapid growth rates, pathogen registance, and desirable timber quality [37, 38].

Herein, we present a highly contiguous, chromosome-level assembly of *C. japonica* generated using a combination of HiFi sequencing, Hi-C scaffolding, and a high-density genetic map. Our chromosome-level assembly of *C. japonica* genome is the first genome sequence, to our knowledge, of a conifer species with high sequence contiguity (N50 contig > 12 Mbp), with most nucleotides mapped to chromosomes (> 97% assigned to the 11 chromosomes, 2n = 22), validated through a high-density genetic map of 7,781 sequence-based markers. Genes were annotated using existing and newly sequenced RNA-seq data, ESTs included in public databases, published Iso-seq data, and homology with proteins of other species, which achieved one of the highest completeness scores (91.4%) of BUSCO [39] among conifer reference genomes [33, 40–43]. This newly sequenced genome will likely serve as a reliable platform for breeding and studying other conifer species as well. For example, it will be valuable for identifying loci associated with tolerance to pathogens, faster growth, adaptation to various environments, and the evolution of conifer genomes and retrotransposons.

## Results

### Genome sequencing and assembly

#### Assembling PacBio HiFi reads

The collective statistics of the PacBio HiFi reads are shown in Table 1. After trimming the first 20 bases of PacBio HiFi reads, assembly by hifiasm [44] resulted in an N50 12-Mbp contig size (Table 1), which is significantly larger than those for published conifer genomes [33]. The total contig size (9.07 Gbp) slightly exceeded the estimated genome size by approximately 200 Mbp based on the GenomeScope k-mer analysis (Additional file 1). We attributed the values of these estimations to residual structural heterozygosity that persisted, despite three rounds of selfing. The algorithm hifiasm assumes that two homologous chromosomes in the target genome may exhibit small variations, such as SNVs or small indels, but are otherwise structurally similar. In cases where this assumption holds, contigs from the two homologous chromosomes were identified, and contigs only from one haplotype were output as primary contigs. When one haplotype contains large structural variations, both haplotypes may be output, yielding a slightly inflated total contig size.

**Table 1:** Summary of sequencing, assembly, and scaffolding statistics for *Cryptomeria japonica* genome

Step	Type	#Reads #Conigs #Scaffolds	Total length (bp)	Average length (bp)	Maximum length (bp)	N50 (bp)	Others
Sequencing	PacBio HiFi	18,877,201	316,631,828,055^$^	16,960	-	17,362	-
Assembly	hifiasm	2,651	9,068,249,119	-	88,939,144	12,036,660	L50: 217
Scaffolding	Hi-C	2,699	9,046,663,964	-	1,007,622,168	754,841,075	Top 11 Scaffolds: 97.6%

^$^ The total length of HiFi sequencing reads reached 36× of *C. japonica* genome

#### Construction of chromosome-length Hi-C scaffolds

**Fig. 1:**
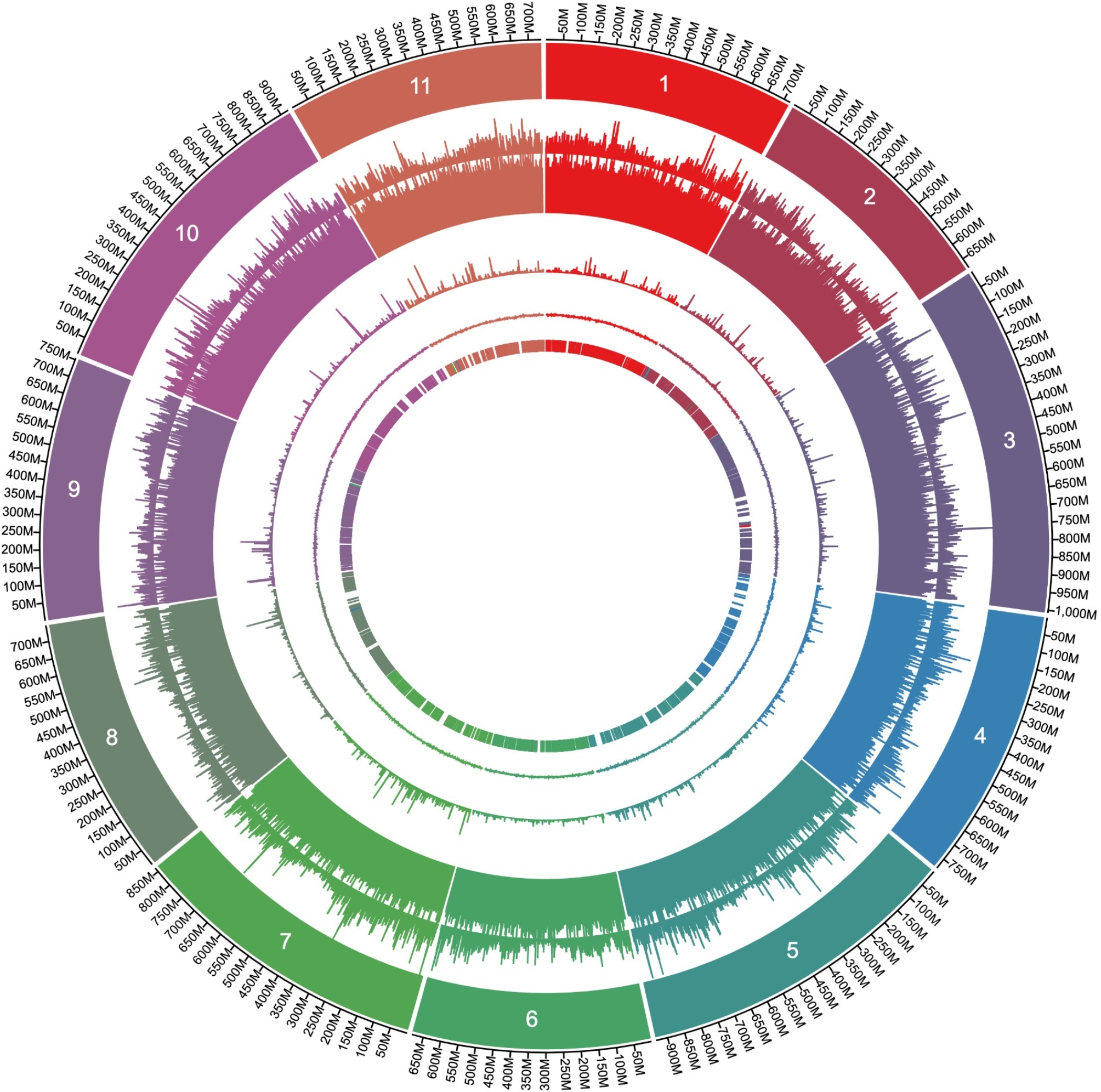
Overview of the 11 chromosomes of the *C. japonica* genome. Rings represent (from the outside) chromosomes, gene density, repeat sequence %, N% (gaps between contigs), GC%, and genetic markers (described later). The genome nucleotide sequence is highly repetitive

Contigs were scaffolded with Hi-C reads aligned to the contigs. Manual review suggested a few necessary corrections for obvious misassemblies. The final chromosome-length scaffolds consisted of the largest 11 scaffolds, corresponding to the 11 chromosomes of *C. japonica*, which contained 97.6% of the nucleotides (Fig. 1; Table 1, and Additional file 2). To further assess the completeness of these chromosome-length scaffolds, telomeric sequences were explored, and it was revealed that 4 of the 11 chromosomes had telomeres at both ends (chr3, chr5, chr7, and chr11), while 4 chromosomes had telomeres at only one end (chr2, chr6, chr8, and chr10) (Additional file 3). These results provide additional insights into the structural completeness of the assembled chromosomes. To further evaluate the completeness and reliability, we employed Merqury [45], which revealed a completeness of 94.95% and a base error rate of 2.12 × 10^–5^%, confirming the high accuracy for a gymnosperm genome assembly.

While the complete chloroplast genome was identified with the length of 131,654 bp, we were unable to reliably located mitochondrial sequences, due to the nature of complex mitochondrial genome structure of conifers [46]. Therefore, potential mitochondrial sequences remained as ordinary scaffolds. The statistics of the final set of scaffold assemblies are presented in Table 1.

#### Genome assembly validation by genetic map

Most markers were consistent with the scaffolds (Additional file 4). The independent development of the genome assembly, the high level of consistency between the Hi-C scaffolds, and the genetic map support the high accuracy of the Hi-C scaffolds through minimizing global misjoins. Multiply mapped markers may be inconsistent, and minor inconsistencies resulting from ambiguities of our merged independent genetic maps are noted (Additional file 4). The mapped markers were well dispersed, and therefore any single locus trait would be easily mapped (Fig. 1). Furthermore, long regions suppressed recombination in the middle of each chromosome. We therefore presumed that these regions corresponded to centromeres and that gene density decreased toward these putative centromeres (Fig. 1).

##### Identifying repeat elements

Similar to other conifer species, the *C. japonica* genome comprised abundant repeat elements, (83.6% of the genome) (Table 2). These repeat elements were uniformly distributed across the genome, showing no observable bias associated with its chromosomal location (Fig. 1). Based on manual inspection of a few randomly selected sequences, we presumed that the majority of “Unknown” and “LTR/Unknown” (included in “Other LTR” in Table 2) was fragmented copies of Gypsy or Copia superfamilies not precisely identified by RepeatModeler2. Moreover, such sequences were shorter than those identified in the Gypsy and Copia genomes.

**Table 2:** Identification of repeat elements. Unknown and LTR/Unknown were presumably fragmented LTR/Gypsy and LTR/Copia that RepeatModeler failed to identify the element category

Class	# bp	% to the contigs
LTR/Gypsy	2,706,211,341	29.8%
LTR/Copia	1,787,198,891	19.7%
LTR/Pao	914,583	0.0%
LTR/ERVK	492,659	0.0%
LTR/Caulimovirus	278,675	0.0%
LTR/ERV1	97,436	0.0%
Other LTR	726,514,838	8.0%
Total LTR	5,221,708,423	57.6%
LINE/L1	266,585,601	2.9%
LINE/L1-Tx1	40,379,039	0.4%
LINE/I	14,549,997	0.2%
LINE/RTE-X	8,624,746	0.1%
LINE/Penelope	2,966,778	0.0%
Total LINE	333,106,161	3.7%
DNA/MULE-MuDR	94,429,850	1.0%
DNA/Sola-2	56,686,823	0.6%
DNA/hAT-Tip100	43,403,967	0.5%
DNA/CMC-EnSpm	32,307,893	0.4%
DNA/hAT-Tag1	27,462,207	0.3%
DNA/TcMar-Fot1	3,788,044	0.0%
DNA/hAT-Ac	2,762,813	0.0%
DNA/Maverick	660,006	0.0%
DNA/Dada	658,721	0.0%
Other DNA	713,765	0.0%
Total DNA	262,874,089	2.9%
RC/Helitron	14,193,395	0.2%
Unknown	1,666,554,795	18.4%
Total interspersed	7,498,436,863	82.7%
Simple repeat	46,895,194	0.5%
rRNA	26,423,944	0.3%
Low complexity	7,191,750	0.1%
Satellite	3,133,394	0.0%
tRNA	562,090	0.0%
Total	7,582,643,235	83.6%

#### Gene and functional annotations

EVidenceModeler [47] integrated the outputs from BRAKER [48] and PASA [47, 49] annotation pipelines, resulting in a set of 152,527 genes, which we designated as a *permissive gene set*, assuming it likely included a substantial number of false-positive genes. As we anticipated, a significant number of predicted genes had little expression evidence (Additional file 5). Since the *permissive gene set* is considered not handy for most types of downstream analysis, we created another gene set by filtering out genes without expression evidence when they have no homology to proteins in other species, after which 55,246 genes (*standard gene set*) remained (Additional file 6). The BUSCO score for the *standard gene set* achieved the highest completeness (91.4%) among conifers [33, 40, 44] for the embryophyta_odb10 conserved gene set (Table 3). Approximately 5% of the duplicated genes (i.e., complete and duplicated) may account for the heterozygosities between the two haplotypes that persisted through three rounds of selfing (see the *Breeding homozygous trees* section). Structural annotation summary (Table 4) revealed the maximum gene and intron length of 1,031,648 bp and 499,149 bp, respectively.

**Table 3:** BUSCO assessment of the assembly (embryophyta_odb10)

Complete %	(single %)	(duplicated %)	Fragmented %	Missing %	Total # genes
91.4	86.1	5.3	3.2	5.4	1,614

**Table 4:** Statistics on structural features of annotated genes

Metric^$^	*Standard gene set*	*Permissive gene set*
#Genes	55,246	152,527
Gene length (bp) Avg	18,765	7,430
Max	1,031,648	1,031,648
CDS length (bp) Avg	263	324
Max	10,881	10,881
Intron length (bp) Avg	6,128	4,016
Max	499,149	499,149
#Genes with introns > 10 kb	9,642	9,724

^$^ Avg and Max represent Average and Maximum, respectively

#### Evolution of LTR retrotransposons

**Fig 2:**
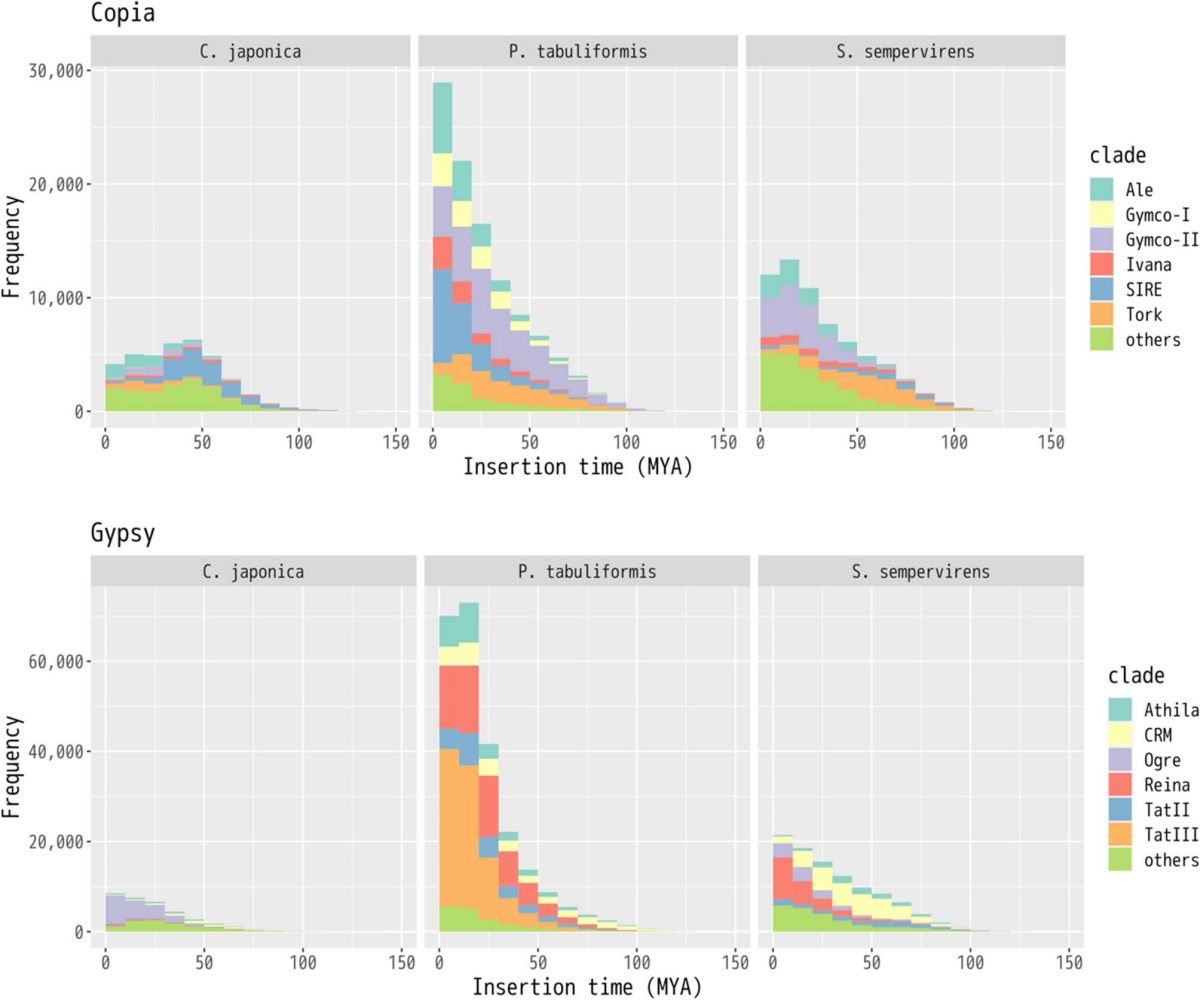
Age distribution of retrotransposons. The Copia (above) and Gypsy (below) superfamilies in the *C. japonica*, *P. tabuliformis*, and *S. sempervirens* genomes, respectively

We identified 37,030 copies of Copia and 34,085 of Gypsy in the *C. japonica* genome, and the families and their statistics (length and insertion time estimates) are shown in Fig. 2 and Additional file 7. There was unambiguous separation between old and corrupting LTR retrotransposons such as SIRE and still-emerging LTR retrotransposons such as Ale and Ogre. Comparing the distribution of LTR retrotransposons in the *C. japonica* genome with that of other conifer species, the *Sequoia sempervirens* and *Pinus tabuliformis* genomes exhibited a significantly different distribution of LTR retrotransposon families. For example, *S. sempervirens* showed few recent insertions, whereas *P. tabuliformis* showed constantly inserted Copia copies. As each species exhibited a unique distribution, more studies are required to characterize the evolutionary history of the LTR retrotransposons in conifer species. To the best of our knowledge, this is the first comparative study of the age distribution of LTR retrotransposons in conifer genomes with an N50 contig size exceeding a megabase. Furthermore, it was possible to largely avoid consideration of the bias that newer LTR retrotransposons with few mutations tended not to be fully reconstructed in a single contig due to assembly errors.

## Discussion

We assembled the *C. japonica* genome sequence with a high degree of continuity (N50 contig: 12.0 Mbp) and therefore were able to map > 97% of the nucleotide sequences to its 11 chromosomes using Hi-C scaffolding and the genetic map. Furthermore, we validated the accuracy of the chromosome-level assembly by comparing it with the high-density genetic map. This allowed us to establish a genetic information platform useful for systematically selecting markers for breeding *C. japonica* in less time. Moreover, we annotated 55,246 genes in the *C. japonica* genome sequence and achieved, to the best of our knowledge, the highest BUSCO score (91.4% complete) among conifers with a chromosome-level assembly. These genome resources thereby reinforce the utility of *C. japonica* as a model conifer tree.

A special subject of interest revealed by the current study was the exceptional diversity of LTR retrotransposons among three conifer species, though those of *C. japonica* and *S. sempervirens* diverged approximately at least 100 million years ago (MYA) during the Mesozoic era [50]. By conducting repeat analysis, including those of species more closely related to *C. japonica*, it may be possible to identify geological events and understand when environmental stress influenced evolution. Moreover, exploring the environmental conditions that triggered the proliferation of LTR transposons is evolutionarily intriguing. It should be noted that acquiring long contiguous genome assemblies is crucial for accurate repeat analysis, as a fragmented genome assembly may lead to the omission of repetitive sequences [51].

The results of the current study also highlighted several distinctive characteristics of LTR-RTs in the three conifer species examined, especially when compared to other plant species. Notably, conifers exhibit a higher proportion of older LTR-RT insertions, with many dating back tens of millions of years, as opposed to the more recent transposon activity commonly observed in other plant species. For example, while the maximum insertion time found in other plant species in the PlantLTRdb database is 5.1 MYA [52], LTR-RTs (SIRE clade in the Copia superfamily) in *C. japonica* have a median insertion time of 143 MYA (Additional file 7), indicating that many of these clades have been present in the genome for an extended evolutionary period. This suggests a distinct evolutionary trajectory for LTR-RTs in conifers, with more ancient insertions compared to the relatively younger transposon activity commonly observed in non-conifer species. Additionally, while the sizes of LTR-RTs in three conifers fall within the general range found in other plants (1,000–25,000 bp) [52], with Gypsy tending to be larger than Copia superfamily, the higher numbers of LTR-RTs in conifers further underscores the importance of these elements in shaping the genomic architecture of conifers with large genomes. These findings suggest that LTR-RTs may have contributed not only to genome size expansion [53, 54] but also to the long-term evolutionary stability of conifer genomes [55, 56].

*C. japonica* has emerged as a valuable model conifer species due to its applicability to advanced technologies such as somatic embryogenesis [11], Agrobacterium-mediated transformation [57], and CRISPR/Cas9 genome editing [58]. These techniques enable the manipulation of diverse traits and facilitate the study of fundamental biological processes, making *C. japonica* a powerful system for conifer research.

For example, the practical application of the *C. japonica* genome sequence is the identification of male-sterile genes [25, 59], which play a significant role in addressing Japanese cedar pollinosis. Male-sterile trees were first discovered in 1992 [60], leading to the initiation of breeding programs aimed to develop male-sterile tree varieties capable of dramatically reducing airborne pollen. Four Mendelian-inherited male sterility loci (*MS1* to *MS4*) were identified through linkage mapping [61], although their sequences are unknown; and the candidate genomic regions for these loci are too large to be sequenced using traditional methods such as bacterial artificial chromosomes (BAC) [62], given the species’ large genome size. To efficiently obtain sequence-based markers for *MS1* to *MS4*, we utilized the genomic sequences of *C. japonica* identified here, employing genetic markers to identify candidate genomic regions for each locus. Our *C. japonica* genome sequences were instrumental in successfully identifying the candidate genes associated with *MS1* and *MS4* [25, 59]. This breakthrough highlights the immense value of our chromosome-level genome sequences to facilitate the identification and characterization of key genes and genetic regions in *C. japonica*.

Leveraging the comprehensive genome sequence and associated functional gene annotations, further research can be conducted to unravel the genetic basis of other economically and ecologically important traits of *C. japonica* [12, 63–66]. A genome-wide association study (GWAS) have detected significant SNPs related to wood properties and reproductive traits in *C. japonica* [63], identifying six significant SNPs and providing novel insights into QTLs related to both traits [65, 67]. Similarly, a GWAS conducted on over 30,000 SNPs from first-generation plus tree genotypes identified markers associated with growth, wood properties, and male fecundity [68]. These markers can now be more precisely linked to functional candidate genes using the chromosome-level genome, enabling targeted trait selection for breeding, and providing valuable insights into the molecular mechanisms underlying these traits in *C. japonica*. Furthermore, comparative genomics studies of *C. japonica* and other conifer species [69] likely will provide insights into the evolutionary relationships and shared genetic networks among conifers, thus contributing to a broader understanding of conifer evolution and gene regulation in forest tree species. Through the identification of conserved gene families and regulatory motifs, the diversity of forest tree species and their ecological functions will be better understood [70, 71], supporting conservation efforts and ecosystem restoration. To fully harness the potential of *C. japonica* as a model conifer, international research collaborations and data sharing are of utmost importance. By establishing collaborative networks among researchers and institutions worldwide, the exchange of research protocols, resources, and knowledge related to *C. japonica* will be facilitated. This collective effort will accelerate the utilization of *C. japonica* as a valuable tool for advancing conifer research and promoting sustainable forest management.

## Conclusions

In this study, the first chromosome-level genome assembly was presented for *Cryptomeria japonica*, a model conifer species of both ecological and economical significance. The integration of HiFi sequencing and Hi-C scaffolding techniques resulted in a highly contiguous genome, overcoming the complexities posed by the species’ large genome size and high repeat content. The resulting assembly, with 97% of the nucleotides mapped to 11 chromosomes, provides a robust framework for advancing genetic studies in conifer species. The detailed annotation of genes and repeat elements compared to other members of the Cupressaceae and Pinaceae families has revealed insights into the evolutionary dynamics of conifers. Additionally, the high completeness of the gene annotation emphasizes the assembly’s utility for both basic biological research and practical breeding programs. This work not only advances our understanding of conifer genome architecture but also establishes a valuable resource for molecular breeding aimed at enhancing traits such as disease resistance, growth rates, and wood quality. The development of this chromosome-level reference genome for *Cryptomeria japonica* has significant implications for conifer genetic research, providing a foundational tool for biotechnological applications and conservation efforts, potentially guiding future efforts in sustainable forestry practices and environmental adaptation studies.

## Methods

### Plant materials

#### Breeding homozygous trees

Assembling highly heterozygous genomes is challenging [32, 72–74], particularly at the time when we initiated our sequencing project of *C. japonica* (e.g., prior to the advent of PacBio HiFi sequencing). To reduce the heterozygous regions of the genome to the extent possible, we attempted to use inbred *C. japonica* trees, which Dr. Masafumi Murai (FFPRI) produced by artificial crossings. However, we were unable to obtain a fully homozygous genome, such as the inbred lines available for rice or maize. Self-fertilization of more than four times was not accomplished. Therefore, for genome sequencing, we selected `Kunisaki-3, I-4-2, S3-3,` a third-generation inbred tree originated from `Kunisaki-3` with the lowest heterozygosity among selfed progenies. The specimen was housed in the xylarium (TWTw-29147) of FFPRI with the voucher. The homozygosity rate of the selected tree was approximately 0.96 as determined using a SNPType assay (Fluidigm) with 158 SNP markers (Additional file 8), which yielded results consistent with those of GenomeScope analysis in the Result section.

### Genome DNA preparation from young leaves

**Fig. 3:**
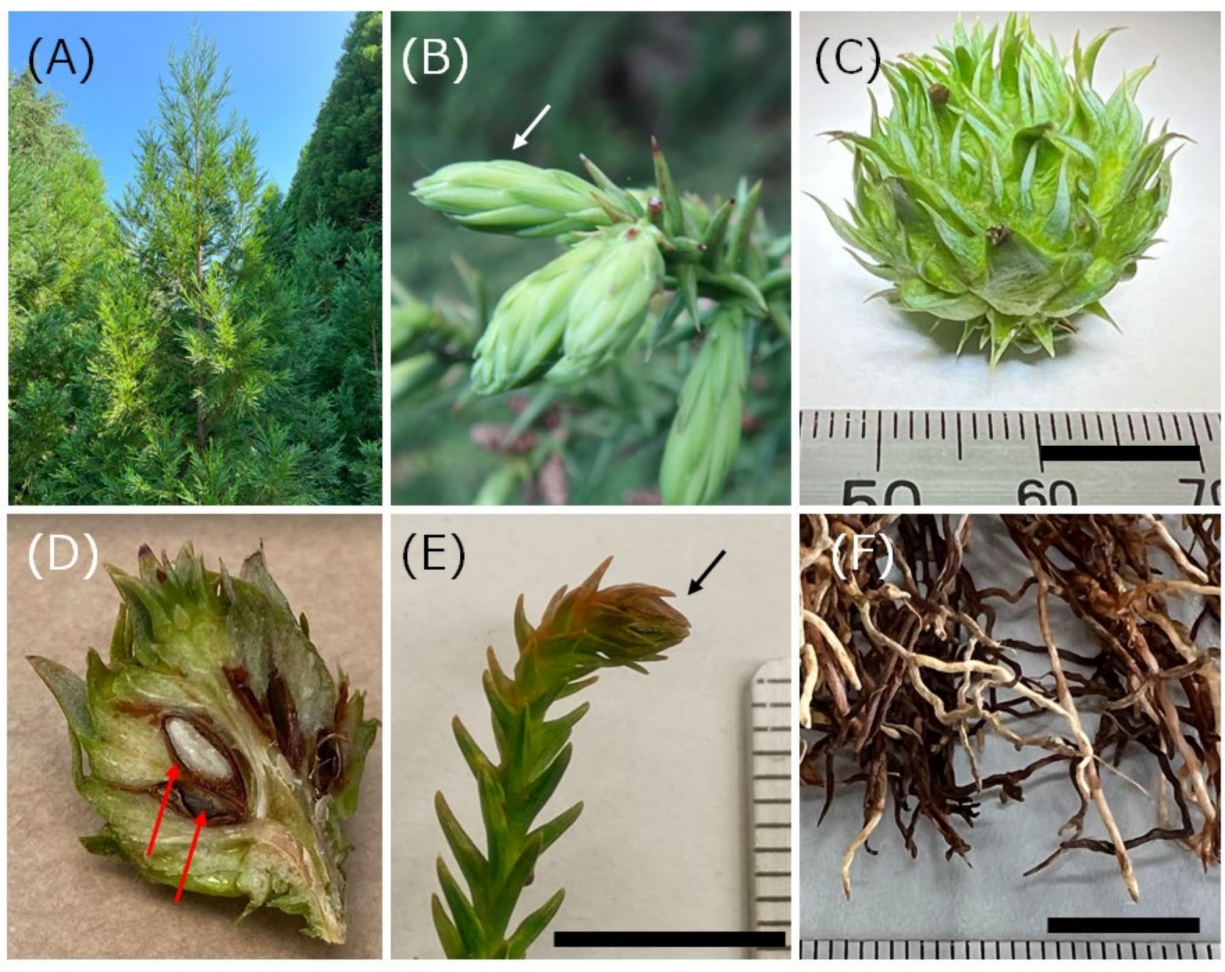
Images of the Japanese cedar (*Cryptomeria japonica*) and tissue samples. **(A)** Overall view of a tree in FFPRI, **(B)** Flushing buds (light-green regions indicated by the white arrow) used for DNA extraction, **(C)** Cone, **(D)** Seeds (red arrows) in a cone cut with a knife. Seeds were removed from the cone for RNA extraction. The white region is a longitudinal section of the seed, representing the megagametophyte and the embryo. **(E)** A female flower (black arrow) on January 11, 2022, **(F)** Roots. Black bars in **(B)**, **(E)**, and **(F)** correspond to 1 cm

Current year shoots (flushing buds) (Fig. 3) were sampled on April 29, 2016, May 9, 2016, and May 9, 2017 from `Kunisaki-3, I-4-2, S3-3.` To prepare high molecular weight (HMW) DNA from *C. japonica*, the leaf sample ground into powder in liquid nitrogen (LN) was mixed with 50 mL of prewash buffer (50 mM Tris-HCl (pH 8.0), 10 mM EDTA, 0.2% 2-mercaptoethanol, 0.3% TritonX) and incubated at room temperature for 1 h with gentle agitation. This prewash step served to reduce the viscosity of the DNA solution. Following centrifugation at 9,000 × *g* for 20 min at 4 °C, the pellet was snap-frozen. Frozen powdered QIAGEN G2 buffer (50 mL) (Qiagen, Netherlands, Cat #1014636), produced by spraying the buffer into LN in a glass beaker, was added to the sample and quickly mixed. After the mixture thawed, 30 µL of RNaseA (QIAGEN, Cat #19101) and 2.5 mL of Proteinase K (QIAGEN, Cat #1019499) were added, and the sample was incubated at room temperature for 2-h without agitation. The sample was centrifuged at 9,000 × *g* for 20 min at 4 °C, and the DNA was extracted from the supernatant using two QIAGEN Genomic-tip 100/G columns (Cat #10243). Genomic DNA was eluted with 5 mL of Buffer QF, precipitated with isopropyl alcohol, and dissolved in 600 µL of TE at 4 °C for a week, yielding 247 µg of DNA (412 ng/µL). We repeated this procedure to obtain another batch of HMW genome DNA from the young leaves of the same tree, yielding 356 µg of DNA (594 ng/µL).

The size distribution of the HMW DNA was evaluated using pulsed-field gel electrophoresis (PFGE). Briefly, 20–200 ng of HMW DNA was electrophoresed through a 1% agarose gel (Seakem Gold Agarose, Lonza, Rockland, ME, USA, Cat #50150) in 0.5× TBE, using the Bio-Rad CHEF Mapper system (Bio-Rad, Hercules, CA, USA, Cat #M1703650), for 15 h; and the DNA in the gel was stained with SYBR Gold dye (Thermo Fisher Scientific, Waltham, MA, USA, Cat #11494). Lambda and 5-kbp ladders (Bio-Rad, Cat #170–3624) were used as size standards (Additional file 9). These results demonstrated that the HMW DNA was approximately 50 kb. The DNA concentration was measured using a Qubit fluorometer (Thermo Fisher Scientific, Cat #Q32866) and a Qubit dsDNA BR Assay Kit (Thermo Fisher Scientific, Cat #Q32850). The quality of the extracted DNA was evaluated by analyzing the absorbance spectrum with a Nanodrop ND-2000 C (Thermo Fisher Scientific).

### NGS library preparation and sequencing

#### PCR-free Illumina read sequencing

The PCR-free Illumina library preparation employed 6 µg of genomic DNA (gDNA) extracted using the QIAGEN Genomic-tip 100/G columns, following the method described in the previous section. The fragmented gDNA ranged in size from 200 to 800 bp with a peak at 550 bp. The settings of a Covaris S2 Focused-ultrasonicator system were as follows: intensity = 4, duty cycle = 10%, cycles per burst = 200, time = 55 s, and temperature = 7 °C. Subsequently, ∼ 400-bp fragments were size-selected by using BluePippin and a 2% EF gel using the tight mode setting. The fragments were subsequently concentrated using an AMpure XP (1.8× vol), yielding 226 µg of DNA with a sharp peak at 410 bp, as determined using an Agilent 2100 Bioanalyzer. A library for whole genome sequencing was prepared using a TruSeq DNA PCR-Free Library Prep Kit (Illumina) according to the manufacturer’s instructions. The DNA concentration of the library was quantified using a KAPA Library Quantification Kit and an Applied Biosystems 7500 Real-Time PCR Systems (Applied Biosystems). The Illumina library was sequenced using the HiSeq X platform (Illumina) at Macrogen Japan (Tokyo, Japan) with the 2 × 151 bp paired-end sequencing protocol. The number of the raw Illumina paired-end reads was 734,439,281.

#### HiFi long-read sequencing

HMW genomic DNA was sheared to an average target size of 20–30 kbp via centrifugation in a Covaris g-Tube (Covaris, Cat #520079) at 2,800–4,800 rpm for 2 min using a microcentrifuge (himac CG15RX). SMRTbell libraries for sequencing using the PacBio Sequel platform were constructed according to the manufacturer’s recommended protocol (HiFi SMRTbell Libraries using SMRTbell Express Template Prep Kit 2.0 PN101-853-100 Version 03 [January 2020] or Version 04 [April 2021]) with minor modifications. We altered the conditions of genome shearing with the g-Tube and the size selection of the library using the BluePippin system (Sage Science, MA, USA) to construct libraries with longer inserts. Specifically, we constructed seven SMRTbell libraries using different centrifugation speeds (4,800 rpm, 3,800 rpm, 4,300 rpm, 4,800 rpm, 3,300 rpm, 2,800 rpm, and 2,800 rpm), yielding libraries with average DNA sizes of 20 kb, 20 kb, 18 kb, 16 kb, 22 kb, 25 kb, and 25 kb, respectively. These insert size distributions were determined using PFGE with the Bio-Rad CHEF Mapper system as previously performed for HMW genome evaluation (see above). These SMRTbell libraries were sequenced in one SMRT cell (movie capture time, 20 h) on a Sequel instrument at the NIBB Functional Genomics Facility and ten cells (movie capture time, 30 h) using a PacBio Sequel II instrument at the National Institute of Genetics, ROIS, Japan (Additional file 10).

#### Hi-C sequencing

The Omni-C library was prepared using a Dovetail Omni-C Kit (Dovetail, Cat #21005) according to the manufacturer’s protocol (Non-mammalian Samples Protocol version 1.0) for plants with modifications. We were unable to obtain chromatin through the standard in-place fixation procedure from leaves, and we therefore employed a third-party protocol [75] to isolate nuclei from leaf samples subjected to nuclease digestion. The ends of chromatins were polished and ligated to a biotinylated bridge adapter prior to proximity ligation of adapter-containing ends. Following proximity ligation, crosslinks were cleaved, DNA was purified to remove proteins, and size-selected. The purified DNA underwent end-repair, ligation to an adapter, and treatment with USER Enzyme. Four sequencing libraries were generated using Illumina-compatible adapters, and biotin-containing fragments were isolated using streptavidin beads before PCR enrichment of the library. These libraries were sequenced on two lanes of the Illumina HiSeq X platform, yielding 470 million 2 × 151 bp read pairs.

### Transcriptome sequencing

To perform gene annotation for *C. japonica*, we carried out RNA-Seq for root, cone (excluding seeds), female flower bud, and seeds in a cone (Fig. 3 and Additional file 11). Cones and female flower buds were sampled from a cutting of `YI96,` obtained by crossing `Yabukuguri` × `Iwao` [64, 76], while roots were sampled from an unidentified seedling obtained from a nursery dealer. Each tissue sample (50–100 mg) was disrupted in LN, and the tissue powder was emulsified with 600 µL of CTAB extraction buffer (Additional file 12) and incubated for 10 min. at 65 °C. After centrifugation for 5 min at 15,000 rpm at 4 °C, the supernatant (400 µL) was mixed with 200 µL of Lysis Buffer of the Maxwell RSC Plant RNA Kit (Promega). RNA was then extracted using a Maxwell RSC Instrument (Promega) according to the manufacturer’s instructions. The extracted RNA was sequenced by Macrogen (Tokyo Japan) to construct sequencing libraries, which were sequenced using a NovaSeq6000 (Illumina) in 2 × 101 bp paired-end form. Published RNA-Seq data (Additional file 11) [77, 78] were also employed.

### Genome size estimation

The genome size was estimated by counting the k-mers in the PCR-free Illumina reads. We used Jellyfish 2.3.0 [79] to count the coverages of 19-mers in the reads and to generate a histogram of the k-mer frequency at each level of coverage. The genome comprised highly repetitive k-mers, and the upper bound of the coverage increased to 10^8^ (jellyfish histo -h 10,000,000). Model fitting using GenomeScope (commit 47e86a1 in GitHub) [80] predicted an approximately 8.9 Gbp haploid size (Additional file 1). Flow cytometry predicted the genome size of *C. japonica* as 22.09 pg/2 C (corresponding to approximately 11 Gbp) using *Hordeum vulgare* cv. Minorimugi as a standard [81]. However, the estimated genome size of *H. vulgare* varies largely among studies (7.27–18.31 pg/2 C) [82, 83]. Moreover, although the flow cytometric analysis [81] adopted the value described in [84] as the genome size of *H. vulgare* cv. Minorimugi, the original estimation is based on *H. vulgare* cv. Sultan, a different cultivar. There is a high level of uncertainty in the genome size estimation of *H. vulgare* compared with that of *C. japonica*. We therefore concluded that the estimated genome size (8.9 Gbp) based on the k-mer analysis is more reliable than the previous estimate (11 Gbp).

### Removal of PacBio HiFi adapters and assembly

Initial quality assessment using fastp v. 0.20.0 [85] revealed that at least the first 12 bases occasionally contained adapter sequences that were not filtered by the official PacBio pipeline (Additional file 13). Trimming the first 20 base pairs eliminated base-frequency bias associated with these adapters (Additional file 14). To evaluate the impact of remaining adapter sequences on assembly, we generated three sets of the HiFi reads by trimming the first 0, 20, and 30 base pairs using fastp v. 0.20.0 [85]. We subsequently attempted the assembly of the three sets of the PacBio HiFi reads using hifiasm ver. 0.16.0-r369 [44] and Flye ver. 2.8.3 [86]. Flye’s assembly jobs failed because of insufficient memory (> 300 Gb), and we estimated that the computation times would exceed 62 days, the maximum permitted in our shared computing environment. In contrast, hifiasm successfully completed the assembly tasks within two days. Hence, we used the output of hifiasm in the downstream analyses (Additional file 15). We selected the assembly derived from reads with a trim size = 20 bp generated by fastp (with options: -t 12 -f 20 -A -V -Q -L -G), because it exhibited the longest N50 contig size, along with other metrics comparable to alternate assemblies.

### Hi-C scaffolding

We utilized Juicer v1.6 [87] and BWA v0.7.17-r1188 [88, 89] to align Hi-C reads to the contigs, subsequently utilizing the 3D-DNA pipeline v180922 [87, 90] to correct misjoins and order, and to position the contigs into scaffolds. We subsequently employed Juicebox Assembly Tools v2.13.07 [90, 91] for manual review of the scaffolds. The 3D-DNA pipeline was subsequently utilized to rescaffold the final chromosome-length scaffolds, and all computational tools were executed using default parameters. We identified and corrected misassemblies based on irregular contact patterns observed in the Hi-C data. Assuming the Hi-C data to be accurate, these patterns strongly indicated assembly errors, which we manually corrected.

### Postprocessing Hi-C scaffolds

Utilizing NCBI-BLAST 2.13.0+ [92] with the parameters specified in VecScreen (as of Dec. 22, 2022) [93], we screened for contaminating sequences such as PacBio adapters within the scaffolds and subsequently masked them as “N”s. Any scaffold whose entire sequence was masked was subsequently removed. The quality and completeness for scaffold sequences were assessed with Merqury v1.3 [45] and Meryl v1.3 [45] with k-mer set to 21.

The scaffold numbers were adjusted to match the corresponding linkage group numbers for *C. japonica* [61, 76, 94]. Only the scaffolds corresponding to linkage groups were renamed from HiC_scaffold_ to chr to reflect their chromosome-level assembly.

To identify scaffolds corresponding to the chloroplast, an existing reference chloroplast sequence (accession number: AP009377) was searched against the scaffolds with BLAST 2.13.0+ (blastn with default parameters) [92]. Subsequent manual review identified scaffolds that largely aligned with the entire reference sequence. A single representative chloroplast sequence was then selected from the assembly, reverse complemented and rotated to improve the alignment with the existing reference sequence, the annotation of which was transferred to the new sequence using RATT [95] (contained in PAGIT V1.64 bit [96]), and designated as chrCh.

### Genome assembly validation by genetic map

A linkage map was constructed from four existing mapping families (F1O7, S1-2, S5HK7, and S8HK5) [61] using LPmerge ver. 1.7 [97] (Additional file 16). The sequence-based markers in the merged genetic map were aligned to the scaffolds using minimap2 ver. 2.17-r941 [98] with the options ​​-c -k 9.

### Identifying repeat elements

We implemented RepeatModeler 2.0.3 [99] to identify repeat elements in the contigs, yielding a repeat library (Table 5) that was subsequently input to RepeatMasker 4.1.2-p1 [100] for identifying repeat elements in the genome. We used the most sensitive preset, -s option. We used the search engine Cross_match [101] of RepeatMasker, because it is the most sensitive engine.

**Table 5:** RepeatModeler2 data

# sequences	Total length	Min. length	Avg. length	Max. length	N50
4,089	10,847,836	34	2,653	39,182	5,201

To specifically detect telomeric repeat sequences, we used quarTeT v1.2.1 (TeloExplorer) [102] with the option -c plant to optimize for plant telomeric repeats. This tool was applied to identify telomeric sequences at the ends of scaffolds to assess the structural completeness of the assembled genome.

### Gene and functional annotations

We used BRAKER 2.1.6 [48] to predict two sets of genes, one based on RNA-seq data and the other on those encoding proteins with homology to protein databases. For the former prediction set, the RNA-seq alignments were used for training the gene model, while the homology-based prediction was conducted using the proteins at the Viridiplantae level of OrthoDB v10 [103–105]. We assembled the RNA-seq data *de novo* using Trinity 2.13.2 [106]. We further performed reference-based transcriptome assembly using StringTie 2.1.7 [107] with the RNA-seq data, ESTs in GenBank/EMBL/DDBJ [77], and published Iso-seq reads [78]. Subsequently, the *de novo* transcriptome assembly, the ESTs, the Iso-seq reads, and the gene structures produced by StringTie were included into the alignment assembly pipeline of PASA 2.5.2 [47, 49] to generate an integrated gene structure on the contigs. Finally, EVidenceModeler 1.1.1 [47] was employed to integrate all the gene sets from the BRAKER pipeline and the PASA alignment assembly pipeline with the following weight combination: BRAKER gene set based on RNA-seq = 1, BRAKER gene set based on protein database = 1, and PASA = 10. We removed genes from the integrated gene set that overlapped 95% of their lengths with repeat elements that typically encode proteins almost entirely within transposable elements. We then eliminated predicted genes that lack expression evidence and homology to those of other species. More specifically, we eliminated genes that lacked both at least 5% overlap with PASA transcripts and homology to proteins in at least one of UniProtKB (release 2022_02) [108], NCBI RefSeq (release 22-06-04) [109], or Viridiplantae in OrthoDB v10 [104], as determined by statistically significant hits using the default parameters of DIAMOND 2.0.15 [110].

We used EnTAP 0.10.8 [111] for functional annotation with default parameters and the databases UniProtKB release 2022_05 [108] and NCBI RefSeq plant proteins release 215 [109]. The descriptions of the top protein hits were transferred to the gene descriptions when EnTAP found it informative. When the hit was not informative, we transferred the EggNOG 4.1 [112, 113] description when available.

Gene prediction and annotation of the genome were initially applied to the hifiasm-based contigs and were subsequently superimposed upon chromosome-level Hi-C-based scaffolds using CrossMap v0.6.3 [114]. We evaluated the quality and completeness of our gene annotations using BUSCO v5.3.0 [39, 115, 116].

### Evolution of LTR retrotransposons

Assembling genomes with abundant repeat elements formerly yielded contigs with less contiguity (i.e., orders of magnitude smaller N50 contig size) when PacBio HiFi reads were not used for genome assembly [117]. Such genome assemblies tended to selectively miss repeat elements, likely leading to their inaccurate analysis. The N50 contig size of our *C. japonica* genome assembly was > 10 Mbp. Thus, the assembly likely may contain more full-length repeat elements than previous conifer genome assemblies, presenting us with an opportunity to more accurately analyze how repeat elements evolved in this gigantic genome. Herein, we analyzed the age distribution of LTR retrotransposons of the *C. japonica* genome.

We first identified the locations of full-length LTR elements in the *C. japonica* genome by running EDTA v2.0.0 [118] with default parameters, and its output contained several putative duplicates that we deduplicated. When a pair of LTR retrotransposon records overlap the left and right LTR ranges, and when the sum of nonoverlapping bases is < 50 bp, the pair is regarded as a duplicate that actually represents a single occurrence of the LTR retrotransposon such that only the lexicographically smallest record was retained.

The evolutionary dynamics of LTR retrotransposons were investigated, primarily following the methodology described by Natali et al. [119]. Given the molecular mechanisms where the sequences of the LTR pairs should be completely identical upon insertion into the genome where they accumulate mutations over time, these sequence divergences between the two LTR copies serve as a molecular clock. For each copy of a candidate LTR retrotransposon, we aligned the pair of the LTR sequences using EMBOSS stretcher 6.6.0.0 [120] to find the discrepancies between them, after which the time distance between the pair of the LTR sequences was estimated using EMBOSS distmat 6.6.0.0 [120] with the Kimura Two-Parameter distance [121]. Using a mutation rate of $\:0.59\times\:1{0}^{-9}$/site/year previously reported for *C. japonica* [122], we estimated the age of the LTR retrotransposon copy. Lastly, we classified each transposon copy with TEsorter ver. 1.3 [123] using Viridiplantae v3.0 of REXdb [124] with the option -db rexdb-plant to indicate the family name. We also conducted the same analysis for *P. tabuliformis* [33] and *S. sempervirens* [44] for comparison, providing insights into the evolutionary dynamics of conifer species.

### ForestGEN genome browser

The genome sequence of *C. japonica* can be explored using ForestGEN’s databases, FORest EST and GENome, where JBrowse ver. 1.16.11 [125] displays the genomic sequences and gene annotations (https://forestgen.ffpri.go.jp/en/info_sugi1.html). ForestGEN offers database functions that enable users to query the functional annotations of predicted genes.

## Electronic supplementary material

Below is the link to the electronic supplementary material.

Supplementary Material 1: **Fig. 1**. Histogram of k-mer frequency at each coverage (k = 19) and model fitting using GenomeScope. Key parameters estimated from the profile are annotated at the top, indicating the genome length (len: 8,893,802,355 bp), unique sequence percentage (uniq: 22.9%), heterozygosity (het: 0.246%), k-mer coverage (kcov: 10.5), error rate (err: 0.209%), and duplication rate (dup: 0.33%)

Supplementary Material 2: **Fig. 2**. Final contact map of the Hi-C analysis after the manual correction. The chromosomal interactions within a genome are shown, as determined using Hi-C analysis. The matrix displays eleven distinct squares along the diagonal, corresponding to the eleven chromosomes of *C. japonica*. Each chromosome appears as a square along the diagonal, because Hi-C contacts were the most frequent within the same chromosome.

Supplementary Material 3: **Table 1**. Telomere identification results for chromosome-length (chr) and Hi-C scaffolds in *Cryptomeria japonica*. The result of telomere identification performed by quarTeT v1.2.1 (TeloExplorer) is described, listing the chromosome or Hi-C scaffold IDs and status indicating whether telomeres were found at both ends (both), only one end (left or right), or not detected (no). For Hi-C scaffolds, only those with telomeres at either or both ends are listed, and those with no identified telomeres are not included.

Supplementary Material 4: **Fig. 3**. Relationship between the marker positions (cM) and the physical position (Mbp) of the markers. The genetic map and the scaffolds are highly consistent, except for the multi-mapped markers (blue dots) where the sequence aligns to multiple locations on the genome.

Supplementary Material 5: **Fig. 4**. Relationship between overlap ratio with PASA assembly and the number of genes. A substantial fraction of predicted genes has little overlap ratio with PASA assembly transcripts. By selecting genes of ≥ 5% overlap with PASA assembly, we remove putative false-positive genes while retaining genes with expression evidence.

Supplementary Material 6: **Table 2**. Gene evidence table. Gene ID: A unique identifier for each gene within SUGI genome annotation version 1; Standard Gene Set: The boolean attribute indicates whether the gene is a part of the *standard gene set*; PASA Overlap: Denotes the presence of overlapping transcripts from PASA [47, 49], implying that gene authenticity is supported by RNA-seq evidence; Ortholog: Indicates whether there is homology to proteins in other species (UniprotKB, NCBI RefSeq, and Viridiplantae of OrthoDB v10; Not Entirely Masked Sequence: Reveals if the gene sequence is unambiguously distinct from repeat elements; TEsorter Hit: Suggests whether the gene contains sequences that may be derived from transposable elements, as identified by TEsorter version 1.3 using the following options (with database): -db rexdb-plant -dp2 (Viridiplantae v3.0 of REXdb). TESorter was run with the -dp2 option to identify genes that are highly likely to be retrotransposons.

Supplementary Material 7: **Table 3**. Length and insertion time estimate (MYA: million years ago) for intact Copia and Gypsy identified in three conifer genomes. For each superfamily (Copia and Gypsy) and for reach clade (Ale, Gymco-I, Gymco-II, Ivana, SIRE, Tork, and others in Copia, and Athila, CRM, Ogre, Reina, TatII, TatIII, and others in Gypsy), counts, lengths (minimum, maximum, mean, and median in base pairs), insertion time estimates (minimum, maximum, mean, and median in MYA) are provided.

Supplementary Material 8: **Table 4**. SNP marker genotypes for `Kunisaki-3, I-4-2, S3-3’. Genotyping was performed using the Fluidigm SNPType Assay. The linkage group and map position (cM) are based on the “YI” mapping family [94] for each marker.

Supplementary Material 9: **Fig. 5**. Pulsed-field gel electrophoresis (PFGE) of high molecular weight DNA from *Cryptomeria japonica* flushing buds. Size distribution of high molecular weight (HMW) DNA from *Cryptomeria japonica* samples (labeled 1727 and 1728) was assessed using PFGE. HMW DNA samples ranging from 20 to 200 ng were electrophoresed through a 1% agarose gel over a 15 h period using the Bio-Rad CHEF Mapper system. A lambda ladder and a 5-kbp ladder (labeled as λ and 5 kb, respectively) were employed as molecular size standards that flanked the lanes containing the HMW DNA samples. Orange lines corresponding to the bands of the size standards (in kbp).

Supplementary Material 10: **Table 5**. Detailed sequencing statistics. Sequencing statistics for each run of PacBio sequencer.

Supplementary Material 11: **Table 6**. List of *C. japonica* RNA-Seq/EST libraries. Each entry is cataloged with a unique ID and linked to the DDBJ Biosample/Experiment/Run accession numbers.

Supplementary Material 12: **Table 7**. Composition of CTAB buffer used to extract RNA from *C. japonica*. List of the reagents included in the CTAB buffer, and their concentrations are shown.

Supplementary Material 13: **Fig. 6**. Base composition report of the HiFi reads using fastp (before trimming). The x-axis represents the position in the read ranging from positions 1 to 50,000, and y-axis shows the base content ratios for each nucleotide (A, T, C, and G, represented by pastel yellow, purple, light green, and blue, respectively). The N (red) and GC (black) percentages are shown. The relatively horizontal lines across the read positions after the 20th base for A, T, C, and G bases indicate the absence of significant base composition bias. In contrast, at the beginning of the read up to 20th base may be biased due to artificial sequences.

Supplementary Material 14: **Fig. 7**. Base composition report of the HiFi reads using fastp (after trimming). The x-axis represents the position in the read ranging from 1 to 50,000, and the y-axis shows the base content ratios for each nucleotide (A, T, C, and G, represented by pastel yellow, purple, light green, and blue, respectively). The N (red) and GC (black) percentages are shown. The relatively horizontal lines across the overall read positions suggest that no significant bias in base composition after trimming.

Supplementary Material 15: **Table 8**. Assembly statistics with varying trimming parameters. The assembly of HiFi reads with trimming length of 0 bp, 20 bp, and 30 bp from the start of the read. At trimming size 20 bp, the longest N50 contig size was attained, suggesting the importance of adapter trimming before assembly.

Supplementary Material 16: **Table 9**. Merged genetic map of four families. Linkage maps were merged from four mapping families with the R package LPmerge ver. 1.7, generating a merged map of 7,781 sequence-based markers.

## Data Availability

The raw PCR-free Illumina reads were deposited in the Sequence Read Archive database of the DNA Data Bank of Japan (DDBJ) under accession number DRR427038, the Omni-C Illumina reads under DRR427039, the PacBio HiFi reads under DRR427323 and DRR424423–DRR424432, RNA-Seq Illumina reads under DRR380356–DRR380360, the assembly and gene annotation under BSEH01000001–BSEH01002698. The assembly, gene annotation, genetic map, and BLAST search are available in the ForestGEN database (https://forestgen.ffpri.go.jp/en/info_sugi1.html).

## Abbreviations

BAC: Bacterial Artificial Chromosomes
BLAST: Basic Local Alignment Search Tool
bp: base pair
BUSCO: Benchmarking Universal Single-Copy Orthologs
BWA: Burrows-Wheeler Aligner
CTAB: cetyltrimethylammonium bromide
DDBJ: DNA Data Bank of Japan
EDTA: ethylenediaminetetraacetic acid
EMBOSS: European Molecular Biology Open Software Suite
FFPRI: Forestry and Forest Products Research Institute
Gbp: Gigabase pairs
GC: Guanine-cytosine
Hi-C: High-throughput chromosome conformation capture
HiFi: High fidelity
HMW: High molecular weight
Iso-seq: Isoform sequencing
kbp: kilobase pairs
LTR: Long terminal repeats
Mbp: Megabase pairs
MS: Male sterility
MYA: Million years ago
NCBI: National Center for Biotechnology Information
NIBB: National Institute for Basic Biology
NIG: National Institute of Genetics
PacBio: Pacific Biosciences
PCR: Polymerase Chain Reaction
RNA-seq: RNA sequencing
ROIS: Research Organization of Information and Systems
SMRT: single-molecule real time
SNP: single nucleotide polymorphism
tRNA: transfer RNA

## References

[1] Farjon A. The Kew Review: conifers of the World. Kew Bull. 2018;73:8.

[2] Yang Y, Ferguson DK, Liu B, Mao K-S, Gao L-M, Zhang S-Z, et al. Recent advances on phylogenomics of gymnosperms and a new classification. Plant Divers. 2022;44:340–50. 35967253 10.1016/j.pld.2022.05.003 PMC9363647

[3] Zabel RA, Morrell JJ. Natural decay resistance (wood durability). In: Zabel RA, Morrell JJ, editors. Wood Microbiology (Second Edition). San Diego: Academic Press; 2020. pp. 455–70.

[4] Vivian MA, Nunes GC, Dobner M Jr, Modes KS, Belini UL. NATURAL DURABILITY OF Cupressus lusitanica, Cryptomeria japonica AND Pinus taeda WOODS IN FIELD TRIAL. Floresta. 2020;50:1603.

[5] Donoso-Fierro C, Becerra J, Bustos-Concha E, Silva M. Chelating and antioxidant activity of lignans from Chilean woods (Cupressaceae). Holzforschung. 2009;63:559–63.

[6] Kusumi J, Tsumura Y, Yoshimaru H, Tachida H. Phylogenetic relationships in Taxodiaceae and Cupressaceae Sensu Stricto based on matK gene, chlL gene, trnl-trnf IGS region, and trnL intron sequences. Am J Bot. 2000;87:1480–8. 11034923

[7] Toda R, VEGETATIVE PROPAGATION IN RELATION TO JAPANESE FOREST TREE IMPROVEMENT. N Z J Forest Sci. 1974;4:410–7.

[8] Ohba K. Clonal forestry with Sugi (Cryptomeria japonica). In: Ahuja M-R, Libby WJ, editors. Clonal forestry II: conservation and application. Berlin, Heidelberg: Springer Berlin Heidelberg; 1993. pp. 66–90.

[9] Aston WG. Nihongi, chronicles of Japan from the earliest times to A.D. 697. London: Kegan Paul; 1896.

[10] Honjyo T. Basic studies on the propagation of Cryptomeria tree based on the grafting. Kyoto Prefect Univ Fac Agr Sci Rep. 1972;24:89–141.

[11] Igasaki T, Sato T, Akashi N, Mohri T, Maruyama E, Kinoshita I, et al. Somatic embryogenesis and plant regeneration from immature zygotic embryos of Cryptomeria japonica D. Don. Plant Cell Rep. 2003;22:239–43. 14586550 10.1007/s00299-003-0687-5

[12] Tsumura Y, Uchiyama K, Moriguchi Y, Kimura MK, Ueno S, Ujino-Ihara T. Genetic differentiation and evolutionary adaptation in Cryptomeria japonica. G3. 2014;4:2389–402. 25320072 10.1534/g3.114.013896 PMC4267934

[13] Rouse RJ, Fantz PR, Bilderback TE. Descriptions and a key to cultivars of Japanese Cedar cultivated in the Eastern United States. Horttechnology. 2000;10:252–66.

[14] Shidei T, Akai T, Ichikawa S. Flower buds formation on Sugi (Cryptomeria japonica) and Metasequoia (Metasequoia glyptosytoboides) by Gibberellic Acid treatment. J JAPANESE FORESTRY Soc. 1959;41:312–5.

[15] Ehrenreich JH, Food and Agriculture Organization of the United Nations. Forestry in China. Food and Agriculture Organization of the United Nations; 1982.

[16] Gil A, Fernández Urrutia M, Isidoro A, Medeiros V, Pacheco J. Sentinel-based Azores Regional Forest Inventory. In: The Ever Growing use of Copernicus across Europe’s Regions: a selection of 99 user stories by local and regional authorities. 2018. pp. 102–3.

[17] Borderes M. The timber of Reunion. Bois Trop. 1991;229:85–94.

[18] Manchester SR, Chen Z-D, Lu A-M, Uemura K. Eastern Asian endemic seed plant genera and their paleogeographic history throughout the Northern Hemisphere. J Syst Evol. 2009;47:1–42.

[19] Tsumura Y. Cryptomeria. In: Kole C, editor. Wild crop relatives: genomic and breeding resources: forest trees. Berlin, Heidelberg: Springer Berlin Heidelberg; 2011. pp. 49–63.

[20] Wu Z, Raven PH, Zhu G. Flora of China illustrations. Volume 4. Cycadaceae Through Fagaceae. Science; 2001.

[21] Cai M, Wen Y, Uchiyama K, Onuma Y, Tsumura Y. Population Genetic Diversity and structure of ancient tree populations of Cryptomeria japonica var. Sinensis based on RAD-seq Data. Forests. 2020;11:1192.

[22] Takahashi M, Miura M, Fukatsu E, Hiraoka Y, Kurita M. Research and project activities for breeding of Cryptomeria japonica D. Don in Japan. J for Res. 2023;28:83–97.

[23] Forestry Agency, Japan. State of Japan’s forests and Forest Management. 2019. https://www.maff.go.jp/e/policies/forestry/attach/pdf/index-8.pdf

[24] Matsubara A, Sakashita M, Gotoh M, Kawashima K, Matsuoka T, Kondo S, et al. Epidemiological survey of allergic Rhinitis in Japan 2019. Nippon Jibiinkoka Gakkai Kaiho. 2020;123:485–90.

[25] Hasegawa Y, Ueno S, Wei F-J, Matsumoto A, Uchiyama K, Ujino-Ihara T, et al. Identification and genetic diversity analysis of a male-sterile gene (MS1) in Japanese cedar (Cryptomeria japonica D. Don). Sci Rep. 2021;11:1496. 33452328 10.1038/s41598-020-80688-1 PMC7810747

[26] Moriguchi Y, Ueno S, Hasegawa Y, Tadama T, Watanabe M, Saito R, et al. Marker-assisted selection of trees with MALE STERILITY 1 in Cryptomeria japonica D. Don. Forests. 2020;11:734.

[27] Watanabe M, Ueno S, Hasegawa Y, Moriguchi Y. Efficient low-cost marker-assisted selection of trees with MALE STERILITY 1 (MS1) in Japanese cedar (Cryptomeria japonica D. Don) using bulk DNA samples. Tree Genet Genomes. 2022;18:29.

[28] Raj Ahuja M, Neale DB. Evolution of genome size in Conifers. Silvae Genet. 2005;54:126–37.

[29] Zonneveld BJM. Conifer genome sizes of 172 species, covering 64 of 67 genera, range from 8 to 72 picogram. Nord J Bot. 2012;30:490–502.

[30] Nystedt B, Street NR, Wetterbom A, Zuccolo A, Lin Y-C, Scofield DG, et al. The Norway spruce genome sequence and conifer genome evolution. Nature. 2013;497:579–84. 23698360 10.1038/nature12211

[31] Wang X-Q, Ran J-H. Evolution and biogeography of gymnosperms. Mol Phylogenet Evol. 2014;75:24–40. 24565948 10.1016/j.ympev.2014.02.005

[32] Mackay J, Dean JFD, Plomion C, Peterson DG, Cánovas FM, Pavy N, et al. Towards decoding the conifer giga-genome. Plant Mol Biol. 2012;80:555–69. 22960864 10.1007/s11103-012-9961-7

[33] Niu S, Li J, Bo W, Yang W, Zuccolo A, Giacomello S, et al. The Chinese pine genome and methylome unveil key features of conifer evolution. Cell. 2022;185:204–e21714. 34965378 10.1016/j.cell.2021.12.006

[34] Lorenz WW, Ayyampalayam S, Bordeaux JM, Howe GT, Jermstad KD, Neale DB, et al. Conifer DBMagic: a database housing multiple de novo transcriptome assemblies for 12 diverse conifer species. Tree Genet Genomes. 2012;8:1477–85.

[35] Nurk S, Koren S, Rhie A, Rautiainen M, Bzikadze AV, Mikheenko A, et al. The complete sequence of a human genome. Science. 2022;376:44–53. 35357919 10.1126/science.abj6987 PMC9186530

[36] Schloissnig S, Kawaguchi A, Nowoshilow S, Falcon F, Otsuki L, Tardivo P et al. The giant axolotl genome uncovers the evolution, scaling, and transcriptional control of complex gene loci. Proc Natl Acad Sci U S A. 2021;118. 10.1073/pnas.2017176118 PMC8053990 33827918

[37] Neale DB, Savolainen O. Association genetics of complex traits in conifers. Trends Plant Sci. 2004;9:325–30. 15231277 10.1016/j.tplants.2004.05.006

[38] Beaulieu J, Doerksen T, Boyle B, Clément S, Deslauriers M, Beauseigle S, et al. Association genetics of wood physical traits in the conifer white spruce and relationships with gene expression. Genetics. 2011;188:197–214. 21385726 10.1534/genetics.110.125781 PMC3120141

[39] Manni M, Berkeley MR, Seppey M, Simão FA, Zdobnov EM. BUSCO Update: Novel and Streamlined Workflows along with broader and deeper phylogenetic Coverage for Scoring of Eukaryotic, Prokaryotic, and viral genomes. Mol Biol Evol. 2021;38:4647–54. 34320186 10.1093/molbev/msab199 PMC8476166

[40] Scott AD, Zimin AV, Puiu D, Workman R, Britton M, Zaman S et al. A reference genome sequence for Giant Sequoia. G3. 2020;10:3907–19. 10.1534/g3.120.401612 PMC7642918 32948606

[41] Xiong X, Gou J, Liao Q, Li Y, Zhou Q, Bi G, et al. The Taxus genome provides insights into paclitaxel biosynthesis. Nat Plants. 2021;7:1026–36. 34267359 10.1038/s41477-021-00963-5 PMC8367818

[42] Song C, Fu F, Yang L, Niu Y, Tian Z, He X, et al. Taxus yunnanensis genome offers insights into gymnosperm phylogeny and taxol production. Commun Biol. 2021;4:1203. 34671091 10.1038/s42003-021-02697-8 PMC8528922

[43] Cheng J, Wang X, Liu X, Zhu X, Li Z, Chu H, et al. Chromosome-level genome of Himalayan yew provides insights into the origin and evolution of the paclitaxel biosynthetic pathway. Mol Plant. 2021;14:1199–209. 33951484 10.1016/j.molp.2021.04.015

[44] Cheng H, Concepcion GT, Feng X, Zhang H, Li H. Haplotype-resolved de novo assembly using phased assembly graphs with hifiasm. Nat Methods. 2021;18:170–5. 33526886 10.1038/s41592-020-01056-5 PMC7961889

[45] Rhie A, Walenz BP, Koren S, Phillippy AM. Merqury: reference-free quality, completeness, and phasing assessment for genome assemblies. Genome Biol. 2020;21:245. 32928274 10.1186/s13059-020-02134-9 PMC7488777

[46] Putintseva YA, Bondar EI, Simonov EP, Sharov VV, Oreshkova NV, Kuzmin DA, et al. Siberian larch (Larix sibirica Ledeb.) Mitochondrial genome assembled using both short and long nucleotide sequence reads is currently the largest known mitogenome. BMC Genomics. 2020;21:654. 32972367 10.1186/s12864-020-07061-4 PMC7517811

[47] Haas BJ, Salzberg SL, Zhu W, Pertea M, Allen JE, Orvis J, et al. Automated eukaryotic gene structure annotation using EVidenceModeler and the program to assemble spliced alignments. Genome Biol. 2008;9:R7. 18190707 10.1186/gb-2008-9-1-r7 PMC2395244

[48] Brůna T, Hoff KJ, Lomsadze A, Stanke M, Borodovsky M. BRAKER2: automatic eukaryotic genome annotation with GeneMark-EP + and AUGUSTUS supported by a protein database. NAR Genom Bioinform. 2021;3:lqaa108. 33575650 10.1093/nargab/lqaa108 PMC7787252

[49] Haas BJ, Delcher AL, Mount SM, Wortman JR, Smith RK Jr, Hannick LI, et al. Improving the Arabidopsis genome annotation using maximal transcript alignment assemblies. Nucleic Acids Res. 2003;31:5654–66. 14500829 10.1093/nar/gkg770 PMC206470

[50] Mao K, Milne RI, Zhang L, Peng Y, Liu J, Thomas P, et al. Distribution of living Cupressaceae reflects the breakup of Pangea. Proc Natl Acad Sci U S A. 2012;109:7793–8. 22550176 10.1073/pnas.1114319109 PMC3356613

[51] Peona V, Weissensteiner MH, Suh A. How complete are complete genome assemblies?-An avian perspective. Mol Ecol Resour. 2018;18:1188–95. 30035372 10.1111/1755-0998.12933

[52] Mokhtar MM, Alsamman AM, El Allali A, PlantLTRdb. An interactive database for 195 plant species LTR-retrotransposons. Front Plant Sci. 2023;14:1134627. 36950350 10.3389/fpls.2023.1134627 PMC10025401

[53] Baniaga AE, Barker MS. Nuclear genome size is positively correlated with median LTR-RT insertion time in fern and lycophyte genomes. Am Fern J. 2019;109:248.

[54] Lee J, Waminal NE, Choi H-I, Perumal S, Lee S-C, Nguyen VB, et al. Rapid amplification of four retrotransposon families promoted speciation and genome size expansion in the genus Panax. Sci Rep. 2017;7:9045. 28831052 10.1038/s41598-017-08194-5 PMC5567358

[55] Zuccolo A, Scofield DG, De Paoli E, Morgante M. The Ty1-copia LTR retroelement family PARTC is highly conserved in conifers over 200 MY of evolution. Gene. 2015;568:89–99. 25982862 10.1016/j.gene.2015.05.028

[56] Cossu RM, Casola C, Giacomello S, Vidalis A, Scofield DG, Zuccolo A. LTR retrotransposons show low levels of unequal recombination and high rates of intraelement gene conversion in large plant genomes. Genome Biol Evol. 2017;9:3449–62. 29228262 10.1093/gbe/evx260 PMC5751070

[57] Taniguchi T, Ohmiya Y, Kurita M, Tsubomura M, Kondo T. Regeneration of transgenic Cryptomeria japonica D. Don after Agrobacterium tumefaciens-mediated transformation of embryogenic tissue. Plant Cell Rep. 2008;27:1461–6. 18542965 10.1007/s00299-008-0569-y

[58] Nanasato Y, Mikami M, Futamura N, Endo M, Nishiguchi M, Ohmiya Y, et al. CRISPR/Cas9-mediated targeted mutagenesis in Japanese cedar (Cryptomeria japonica D. Don). Sci Rep. 2021;11:16186. 34376731 10.1038/s41598-021-95547-w PMC8355236

[59] Kakui H, Ujino-Ihara T, Hasegawa Y, Tsurisaki E, Futamura N, Iwai J, et al. A single-nucleotide substitution of CjTKPR1 determines pollen production in the gymnosperm plant Cryptomeria japonica. PNAS Nexus. 2023;2:gad236. 10.1093/pnasnexus/pgad236 PMC10408704 37559748

[60] Taira H, Teranishi H, Kenda Y. A case study of male sterility in sugi (Cryptomeria japonica). J JAPANESE FORESTRY Soc. 1993;75:377–9.

[61] Hasegawa Y, Ueno S, Matsumoto A, Ujino-Ihara T, Uchiyama K, Totsuka S, et al. Fine mapping of the male-sterile genes (MS1, MS2, MS3, and MS4) and development of SNP markers for marker-assisted selection in Japanese cedar (Cryptomeria japonica D. Don). PLoS ONE. 2018;13:e0206695. 30439978 10.1371/journal.pone.0206695 PMC6237302

[62] Tamura M, Hisataka Y, Moritsuka E, Watanabe A, Uchiyama K, Futamura N, et al. Analyses of random BAC clone sequences of Japanese cedar, Cryptomeria japonica. Tree Genet Genomes. 2015;11:50.

[63] Uchiyama K, Iwata H, Moriguchi Y, Ujino-Ihara T, Ueno S, Taguchi Y, et al. Demonstration of genome-wide association studies for identifying markers for wood property and male strobili traits in Cryptomeria japonica. PLoS ONE. 2013;8:e79866. 24260312 10.1371/journal.pone.0079866 PMC3833940

[64] Mori H, Ueno S, Ujino-Ihara T, Fujiwara T, Yamashita K, Kanetani S, et al. Genotype-by-environment interaction and genetic dissection of heartwood color in Cryptomeria japonica based on multiple common gardens and quantitative trait loci mapping. PLoS ONE. 2022;17:e0270522. 35793335 10.1371/journal.pone.0270522 PMC9258842

[65] Mori H, Ueno S, Ujino-Ihara T, Fujiwara T, Yamashita K, Kanetani S, et al. Mapping quantitative trait loci for growth and wood property traits in Cryptomeria japonica across multiple environments. Tree Genet Genomes. 2019;15:43.

[66] Moriguchi Y, Saito R, Ueno S, Hasegawa Y, Kakui H, Matsumoto A. Localization of TWISTED NEEDLES locus on linkage map of Japanese Cedar (Cryptomeria japonica D. Don). Forests. 2022;13.

[67] Ujino-Ihara T, Iwata H, Taguchi Y, Tsumura Y. Identification of QTLs associated with male strobilus abundance in Cryptomeria japonica. Tree Genet Genomes. 2012;8:1319–29.

[68] Hiraoka Y, Fukatsu E, Mishima K, Hirao T, Teshima KM, Tamura M, et al. Potential of genome-wide studies in unrelated plus trees of a coniferous species, Cryptomeria japonica (Japanese cedar). Front Plant Sci. 2018;9:1322. 30254658 10.3389/fpls.2018.01322 PMC6141754

[69] de Miguel M, Bartholomé J, Ehrenmann F, Murat F, Moriguchi Y, Uchiyama K, et al. Evidence of intense chromosomal shuffling during conifer evolution. Genome Biol Evol. 2015;7:2799–809. 26400405 10.1093/gbe/evv185 PMC4684699

[70] Satake A, Kelly D. Studying the genetic basis of masting. Philos Trans R Soc Lond B Biol Sci. 2021;376:20210116. 34657458 10.1098/rstb.2021.0116 PMC8520782

[71] Gao Y, Liu X, Jin Y, Wu J, Li S, Li Y, et al. Drought induces epitranscriptome and proteome changes in stem-differentiating xylem of Populus trichocarpa. Plant Physiol. 2022;190:459–79. 35670753 10.1093/plphys/kiac272 PMC9434199

[72] Pryszcz LP, Gabaldón T. Redundans: an assembly pipeline for highly heterozygous genomes. Nucleic Acids Res. 2016;44:e113. 27131372 10.1093/nar/gkw294 PMC4937319

[73] Claros MG, Bautista R, Guerrero-Fernández D, Benzerki H, Seoane P, Fernández-Pozo N. Why assembling plant genome sequences is so challenging. Biology. 2012;1:439–59. 24832233 10.3390/biology1020439 PMC4009782

[74] Howe K, Clark MD, Torroja CF, Torrance J, Berthelot C, Muffato M, et al. The zebrafish reference genome sequence and its relationship to the human genome. Nature. 2013;496:498–503. 23594743 10.1038/nature12111 PMC3703927

[75] Workman R, Fedak R, Kilburn D, Hao S, Liu K, Timp W. High Molecular Weight DNA Extraction from Recalcitrant Plant Species for Third Generation Sequencing. 2019.

[76] Moriguchi Y, Uchiyama K, Ueno S, Ujino-Ihara T, Matsumoto A, Iwai J, et al. A high-density linkage map with 2560 markers and its application for the localization of the male-sterile genes ms3 and ms4 in Cryptomeria japonica D. Don. Tree Genet Genomes. 2016;12:57.

[77] Futamura N, Totoki Y, Toyoda A, Igasaki T, Nanjo T, Seki M, et al. Characterization of expressed sequence tags from a full-length enriched cDNA library of Cryptomeria japonica male strobili. BMC Genomics. 2008;9:383. 18691438 10.1186/1471-2164-9-383 PMC2568000

[78] Wei F-J, Ueno S, Ujino-Ihara T, Saito M, Tsumura Y, Higuchi Y, et al. Construction of a reference transcriptome for the analysis of male sterility in sugi (Cryptomeria japonica D. Don) focusing on MALE STERILITY 1 (MS1). PLoS ONE. 2021;16:e0247180. 33630910 10.1371/journal.pone.0247180 PMC7935350

[79] Marçais G, Kingsford C. A fast, lock-free approach for efficient parallel counting of occurrences of k-mers. Bioinformatics. 2011;27:764–70. 21217122 10.1093/bioinformatics/btr011 PMC3051319

[80] Vurture GW, Sedlazeck FJ, Nattestad M, Underwood CJ, Fang H, Gurtowski J, et al. GenomeScope: fast reference-free genome profiling from short reads. Bioinformatics. 2017;33:2202–4. 28369201 10.1093/bioinformatics/btx153 PMC5870704

[81] Hizume M, Kondo T, Shibata F, Ishizuka R. Flow Cytometric determination of genome size in the Taxodiaceae, Cupressaceae Sensu Stricto and Sciadopityaceae. Cytologia. 2001;66:307–11.

[82] Marie D, Brown SC. A cytometric exercise in plant DNA histograms, with 2 C values for 70 species. Biol Cell. 1993;78:41–51. 8220226 10.1016/0248-4900(93)90113-s

[83] Pustahija F, Brown SC, Bogunić F, Bašić N, Muratović E, Ollier S, et al. Small genomes dominate in plants growing on serpentine soils in West Balkans, an exhaustive study of 8 habitats covering 308 taxa. Plant Soil. 2013;373:427–53.

[84] Bennett MD, Smith JB, Heslop-Harrison JS, Riley R. Nuclear DNA amounts in angiosperms. Proceedings of the Royal Society of London Series B Biological Sciences. 1997;216:179–99.

[85] Chen S, Zhou Y, Chen Y, Gu J. Fastp: an ultra-fast all-in-one FASTQ preprocessor. Bioinformatics. 2018;34:i884–90. 30423086 10.1093/bioinformatics/bty560 PMC6129281

[86] Kolmogorov M, Yuan J, Lin Y, Pevzner PA. Assembly of long, error-prone reads using repeat graphs. Nat Biotechnol. 2019;37:540–6. 30936562 10.1038/s41587-019-0072-8

[87] Dudchenko O, Batra SS, Omer AD, Nyquist SK, Hoeger M, Durand NC, et al. De novo assembly of the Aedes aegypti genome using Hi-C yields chromosome-length scaffolds. Science. 2017;356:92–5. 28336562 10.1126/science.aal3327 PMC5635820

[88] Li H, Durbin R. Fast and accurate short read alignment with Burrows-Wheeler transform. Bioinformatics. 2009;25:1754–60. 19451168 10.1093/bioinformatics/btp324 PMC2705234

[89] Li H. Aligning sequence reads, clone sequences and assembly contigs with BWA-MEM. arXiv org. 2013;q-bio.GN

[90] Durand NC, Robinson JT, Shamim MS, Machol I, Mesirov JP, Lander ES, et al. Juicebox provides a visualization system for Hi-C contact maps with unlimited zoom. Cell Syst. 2016;3:99–101. 27467250 10.1016/j.cels.2015.07.012 PMC5596920

[91] Dudchenko O, Shamim MS, Batra SS, Durand NC, Musial NT, Mostofa R et al. The Juicebox Assembly Tools module facilitates de novo assembly of mammalian genomes with chromosome-length scaffolds for under $1000. bioRxiv. 2018;:254797.

[92] Camacho C, Coulouris G, Avagyan V, Ma N, Papadopoulos J, Bealer K et al. BLAST+: Architecture and applications. BMC Bioinformatics. 2009;10. 10.1186/1471-2105-10-421 PMC2803857 20003500

[93] NCBI. NCBI VecScreen (https://www.ncbi.nlm.nih.gov/tools/vecscreen/).

[94] Moriguchi Y, Ujino-Ihara T, Uchiyama K, Futamura N, Saito M, Ueno S, et al. The construction of a high-density linkage map for identifying SNP markers that are tightly linked to a nuclear-recessive major gene for male sterility in Cryptomeria japonica D. Don. BMC Genomics. 2012;13:95. 22424262 10.1186/1471-2164-13-95 PMC3386010

[95] Otto TD, Dillon GP, Degrave WS, Berriman M. RATT: Rapid Annotation transfer Tool. Nucleic Acids Res. 2011;39:e57. 21306991 10.1093/nar/gkq1268 PMC3089447

[96] Swain MT, Tsai IJ, Assefa SA, Newbold C, Berriman M, Otto TD. A post-assembly genome-improvement toolkit (PAGIT) to obtain annotated genomes from contigs. Nat Protoc. 2012;7:1260–84. 22678431 10.1038/nprot.2012.068 PMC3648784

[97] Endelman JB, Plomion C. LPmerge: an R package for merging genetic maps by linear programming. Bioinformatics. 2014;30:1623–4. 24532720 10.1093/bioinformatics/btu091

[98] Li H. Minimap2: pairwise alignment for nucleotide sequences. Bioinformatics. 2018;34:3094–100. 29750242 10.1093/bioinformatics/bty191 PMC6137996

[99] Flynn JM, Hubley R, Goubert C, Rosen J, Clark AG, Feschotte C, et al. RepeatModeler2 for automated genomic discovery of transposable element families. Proc Natl Acad Sci U S A. 2020;117:9451–7. 32300014 10.1073/pnas.1921046117 PMC7196820

[100] Smit AFA, Hubley R, Green P. Dec RepeatMasker Open-4.0. https://www.repeatmasker.org/. Accessed 22 2023.

[101] Green P. cross_match. http://www.phrap.org/

[102] Lin Y, Ye C, Li X, Chen Q, Wu Y, Zhang F, et al. quarTeT: a telomere-to-telomere toolkit for gap-free genome assembly and centromeric repeat identification. Hortic Res. 2023;10:uhad127. 37560017 10.1093/hr/uhad127 PMC10407605

[103] Kriventseva EV, Tegenfeldt F, Petty TJ, Waterhouse RM, Simão FA, Pozdnyakov IA et al. OrthoDB v8: update of the hierarchical catalog of orthologs and the underlying free software. Nucleic Acids Res. 2015;43 Database issue:D250-6. 10.1093/nar/gku1220 PMC4383991 25428351

[104] Kriventseva EV, Kuznetsov D, Tegenfeldt F, Manni M, Dias R, Simão FA, et al. OrthoDB v10: sampling the diversity of animal, plant, fungal, protist, bacterial and viral genomes for evolutionary and functional annotations of orthologs. Nucleic Acids Res. 2019;47:D807–11. 30395283 10.1093/nar/gky1053 PMC6323947

[105] Zdobnov EM, Tegenfeldt F, Kuznetsov D, Waterhouse RM, Simão FA, Ioannidis P, et al. OrthoDB v9.1: cataloging evolutionary and functional annotations for animal, fungal, plant, archaeal, bacterial and viral orthologs. Nucleic Acids Res. 2017;45:D744–9. 27899580 10.1093/nar/gkw1119 PMC5210582

[106] Grabherr MG, Haas BJ, Yassour M, Levin JZ, Thompson DA, Amit I, et al. Full-length transcriptome assembly from RNA-Seq data without a reference genome. Nat Biotechnol. 2011;29:644–52. 21572440 10.1038/nbt.1883 PMC3571712

[107] Kovaka S, Zimin AV, Pertea GM, Razaghi R, Salzberg SL, Pertea M. Transcriptome assembly from long-read RNA-seq alignments with StringTie2. Genome Biol. 2019;20:278. 31842956 10.1186/s13059-019-1910-1 PMC6912988

[108] UniProt Consortium. UniProt: the Universal protein knowledgebase in 2023. Nucleic Acids Res. 2022. 10.1093/nar/gkac1052. 10.1093/nar/gkac1052 PMC9825514 36408920

[109] O’Leary NA, Wright MW, Brister JR, Ciufo S, Haddad D, McVeigh R, et al. Reference sequence (RefSeq) database at NCBI: current status, taxonomic expansion, and functional annotation. Nucleic Acids Res. 2016;44:D733–45. 26553804 10.1093/nar/gkv1189 PMC4702849

[110] Buchfink B, Reuter K, Drost H-G. Sensitive protein alignments at tree-of-life scale using DIAMOND. Nat Methods. 2021;18:366–8. 33828273 10.1038/s41592-021-01101-x PMC8026399

[111] Hart AJ, Ginzburg S, Xu MS, Fisher CR, Rahmatpour N, Mitton JB, et al. EnTAP: bringing faster and smarter functional annotation to non-model eukaryotic transcriptomes. Mol Ecol Resour. 2020;20:591–604. 31628884 10.1111/1755-0998.13106

[112] Cantalapiedra CP, Hernández-Plaza A, Letunic I, Bork P, Huerta-Cepas J. eggNOG-mapper v2: functional annotation, Orthology assignments, and Domain Prediction at the Metagenomic Scale. Mol Biol Evol. 2021;38:5825–9. 34597405 10.1093/molbev/msab293 PMC8662613

[113] Huerta-Cepas J, Szklarczyk D, Heller D, Hernández-Plaza A, Forslund SK, Cook H, et al. eggNOG 5.0: a hierarchical, functionally and phylogenetically annotated orthology resource based on 5090 organisms and 2502 viruses. Nucleic Acids Res. 2019;47:D309–14. 30418610 10.1093/nar/gky1085 PMC6324079

[114] Zhao H, Sun Z, Wang J, Huang H, Kocher J-P, Wang L. CrossMap: a versatile tool for coordinate conversion between genome assemblies. Bioinformatics. 2014;30:1006–7. 24351709 10.1093/bioinformatics/btt730 PMC3967108

[115] Simão FA, Waterhouse RM, Ioannidis P, Kriventseva EV, Zdobnov EM. BUSCO: assessing genome assembly and annotation completeness with single-copy orthologs. Bioinformatics. 2015;31:3210–2. 26059717 10.1093/bioinformatics/btv351

[116] Waterhouse RM, Seppey M, Simão FA, Manni M, Ioannidis P, Klioutchnikov G, et al. BUSCO applications from Quality assessments to Gene Prediction and Phylogenomics. Mol Biol Evol. 2018;35:543–8. 29220515 10.1093/molbev/msx319 PMC5850278

[117] Rabanal FA, Gräff M, Lanz C, Fritschi K, Llaca V, Lang M, et al. Pushing the limits of HiFi assemblies reveals centromere diversity between two Arabidopsis thaliana genomes. Nucleic Acids Res. 2022;50:12309–27. 36453992 10.1093/nar/gkac1115 PMC9757041

[118] Ou S, Su W, Liao Y, Chougule K, Agda JRA, Hellinga AJ, et al. Benchmarking transposable element annotation methods for creation of a streamlined, comprehensive pipeline. Genome Biol. 2019;20:275. 31843001 10.1186/s13059-019-1905-y PMC6913007

[119] Natali L, Cossu RM, Mascagni F, Giordani T, Cavallini A. A survey of Gypsy and Copia LTR-retrotransposon superfamilies and lineages and their distinct dynamics in the Populus trichocarpa (L.) genome. Tree Genet Genomes. 2015;11:107.

[120] Rice P, Longden L, Bleasby A. EMBOSS: the European Molecular Biology Open Software suite. Trends Genet. 2000;16:276–7. 10827456 10.1016/s0168-9525(00)02024-2

[121] Kimura M. A simple method for estimating evolutionary rates of base substitutions through comparative studies of nucleotide sequences. J Mol Evol. 1980;16:111–20. 7463489 10.1007/BF01731581

[122] Kusumi J, Tsumura Y, Tachida H. Evolutionary rate variation in two conifer species, Taxodium distichum (L.) Rich. Var. Distichum (baldcypress) and Cryptomeria japonica (Thunb. Ex L.f.) D. Don (Sugi, Japanese cedar). Genes Genet Syst. 2015;90:305–15. 26687861 10.1266/ggs.14-00079

[123] Zhang R-G, Li G-Y, Wang X-L, Dainat J, Wang Z-X, Ou S et al. TEsorter: an accurate and fast method to classify LTR-retrotransposons in plant genomes. Hortic Res. 2022;9. 10.1093/hr/uhac017 PMC9002660 35184178

[124] Neumann P, Novák P, Hoštáková N, Macas J. Systematic survey of plant LTR-retrotransposons elucidates phylogenetic relationships of their polyprotein domains and provides a reference for element classification. Mob DNA. 2019;10:1. 30622655 10.1186/s13100-018-0144-1 PMC6317226

[125] Buels R, Yao E, Diesh CM, Hayes RD, Munoz-Torres M, Helt G, et al. JBrowse: a dynamic web platform for genome visualization and analysis. Genome Biol. 2016;17:66. 27072794 10.1186/s13059-016-0924-1 PMC4830012

