# Supplementary material for: A chromosome-level genome assembly of a model conifer plant, the Japanese cedar, Cryptomeria japonica D. Don
Source: BMC Genomics. 2024 Nov 5;25:1039. doi: 10.1186/s12864-024-10929-4 (PMC11539532; doi:10.1186/s12864-024-10929-4)
Supplement: Supplementary file 1 — Supplementary Material 1: Fig. 1. Histogram of k-mer frequency at each coverage (k = 19) and model fitting using GenomeScope. Key parameters estimated from the profile are annotated at the top, indicating the genome length (len: 8,893,802,355 bp), unique sequence percentage (uniq: 22.9%), heterozygosity (het: 0.246%), k-mer coverage (kcov: 10.5), error rate (err: 0.209%), and duplication rate (dup: 0.33%) [file 12864_2024_10929_MOESM1_ESM.docx]

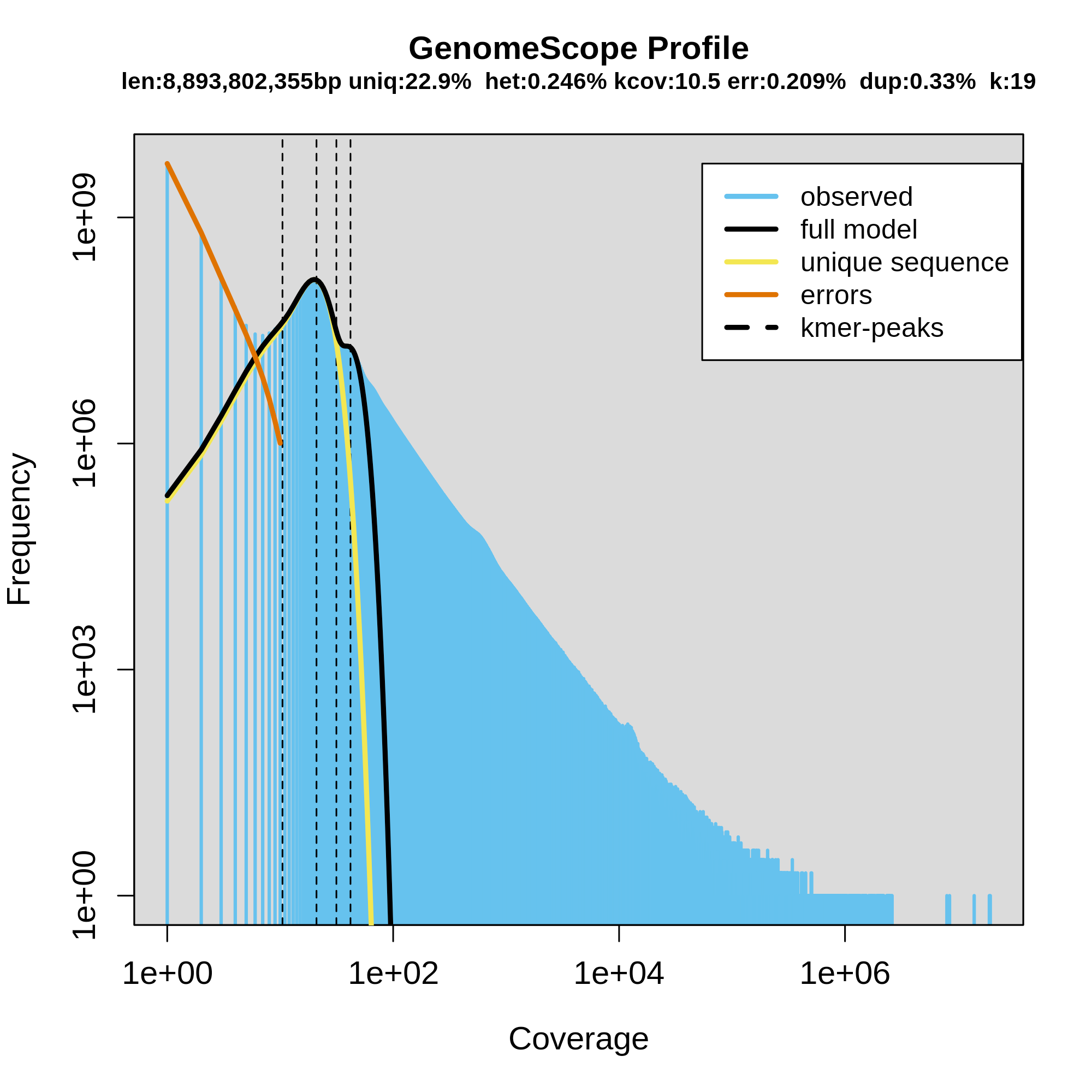


**Supplementary Figure 1** Histogram of k-mer frequency at each coverage (k = 19) and model fitting using GenomeScope.

Key parameters estimated from the profile are annotated at the top, indicating the genome length (len: 8,893,802,355 bp), unique sequence percentage (uniq: 22.9%), heterozygosity (het: 0.246%), k-mer coverage (kcov: 10.5), error rate (err: 0.209%), and duplication rate (dup: 0.33%).
