# Supplementary material for: A chromosome-level genome assembly of a model conifer plant, the Japanese cedar, Cryptomeria japonica D. Don
Source: BMC Genomics. 2024 Nov 5;25:1039. doi: 10.1186/s12864-024-10929-4 (PMC11539532; doi:10.1186/s12864-024-10929-4)
Supplement: Supplementary file 2 — Supplementary Material 2: Fig. 2. Final contact map of the Hi-C analysis after the manual correction. The chromosomal interactions within a genome are shown, as determined using Hi-C analysis. The matrix displays eleven distinct squares along the diagonal, corresponding to the eleven chromosomes of C. japonica. Each chromosome appears as a square along the diagonal, because Hi-C contacts were the most frequent within the same chromosome. [file 12864_2024_10929_MOESM2_ESM.docx]

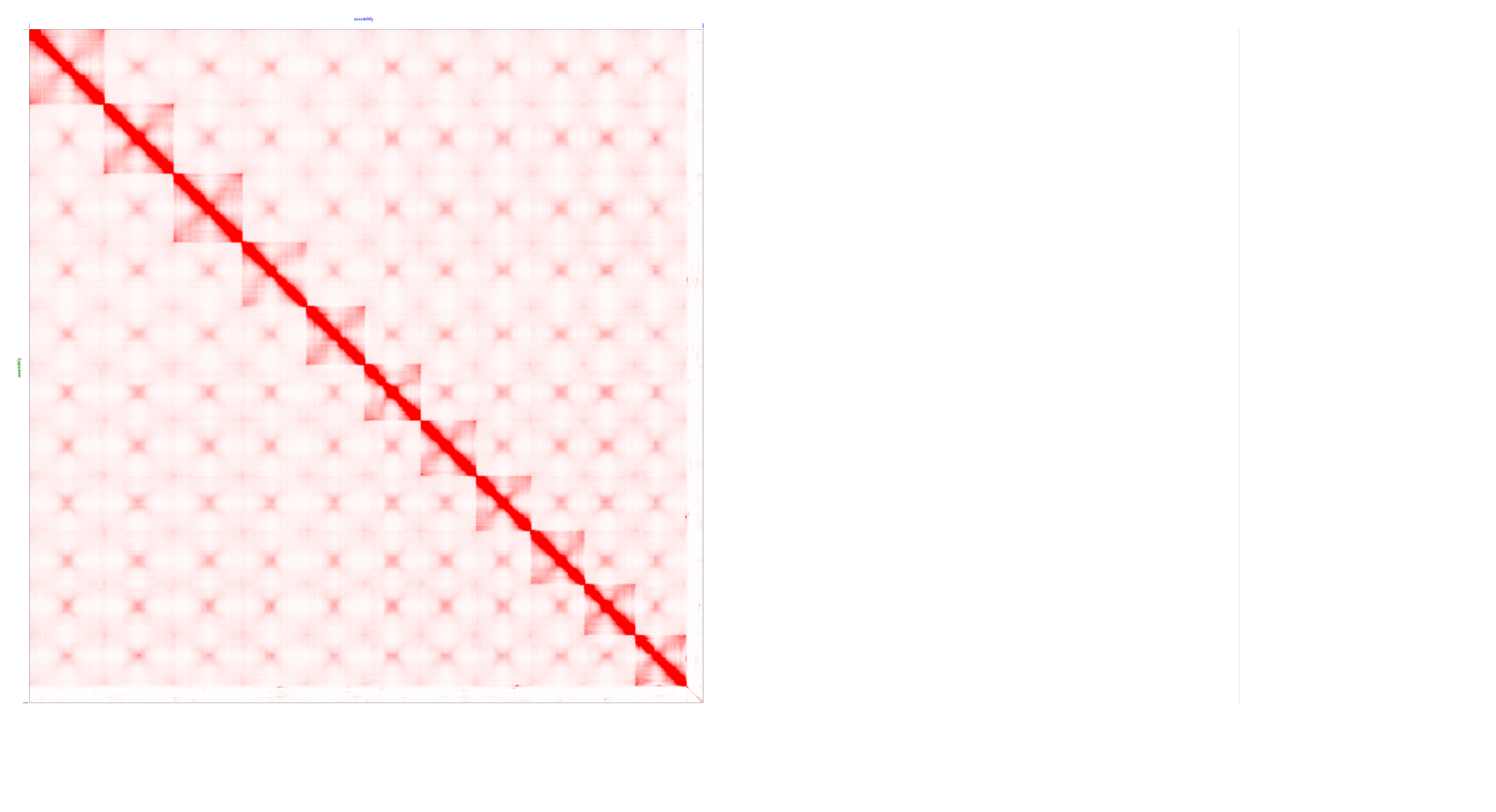


**Supplementary Figure 2** Final contact map of the Hi-C analysis after the manual correction.

The chromosomal interactions within a genome are shown, as determined using Hi-C analysis. The matrix displays eleven distinct squares along the diagonal, corresponding to the eleven chromosomes of *C. japonica*. Each chromosome appears as a square along the diagonal, because Hi-C contacts were the most frequent within the same chromosome.
