# Supplementary material for: A chromosome-level genome assembly of a model conifer plant, the Japanese cedar, Cryptomeria japonica D. Don
Source: BMC Genomics. 2024 Nov 5;25:1039. doi: 10.1186/s12864-024-10929-4 (PMC11539532; doi:10.1186/s12864-024-10929-4)
Supplement: Supplementary file 4 — Supplementary Material 4: Fig. 3. Relationship between the marker positions (cM) and the physical position (Mbp) of the markers. The genetic map and the scaffolds are highly consistent, except for the multi-mapped markers (blue dots) where the sequence aligns to multiple locations on the genome. [file 12864_2024_10929_MOESM4_ESM.docx]

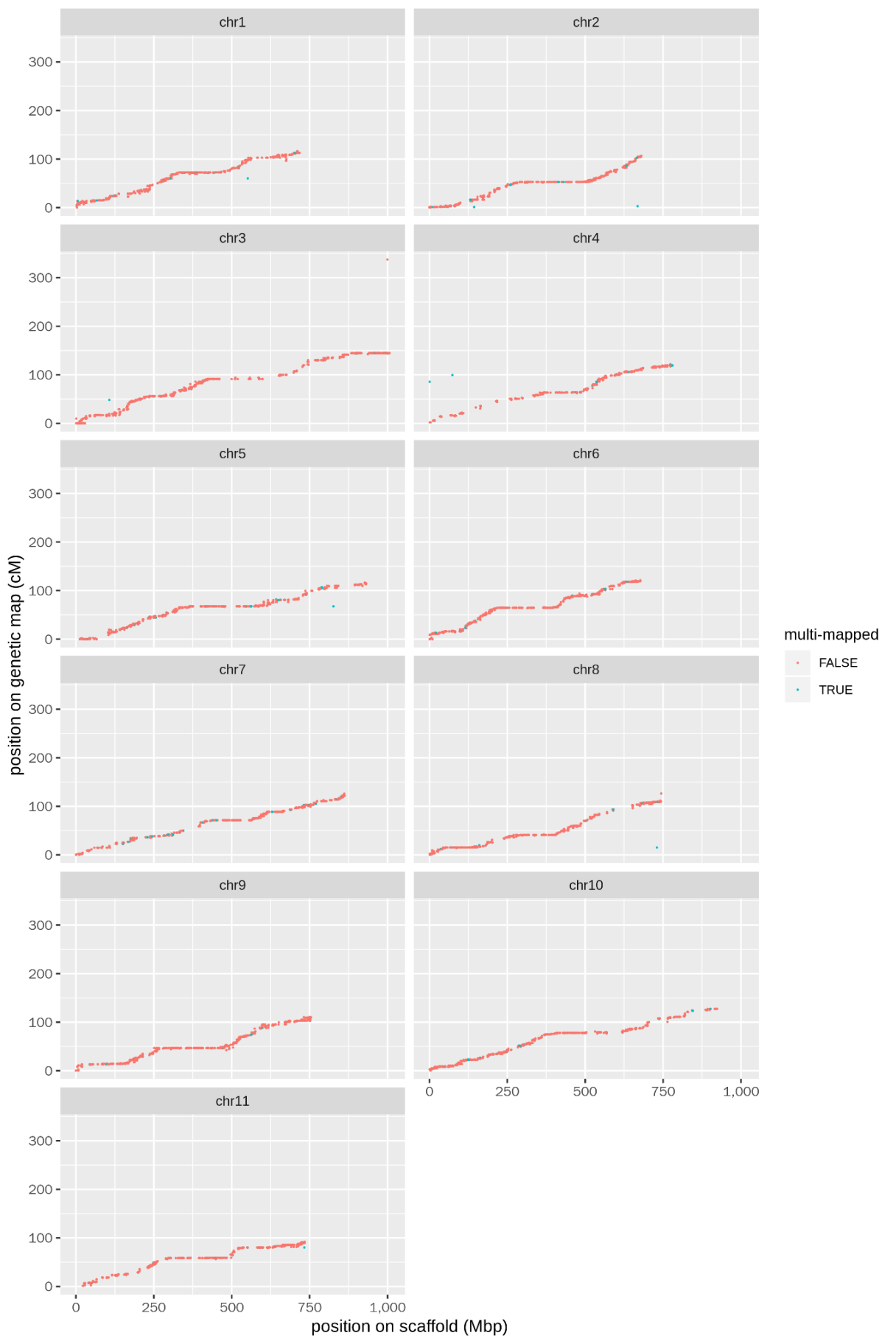


**Supplementary Figure 3** Relationship between the marker positions (cM) and the physical position (Mbp) of the markers.

The genetic map and the scaffolds are highly consistent, except for the multi-mapped markers (blue dots) where the sequence aligns to multiple locations on the genome.
