# Supplementary material for: A chromosome-level genome assembly of a model conifer plant, the Japanese cedar, Cryptomeria japonica D. Don
Source: BMC Genomics. 2024 Nov 5;25:1039. doi: 10.1186/s12864-024-10929-4 (PMC11539532; doi:10.1186/s12864-024-10929-4)
Supplement: Supplementary file 5 — Supplementary Material 5: Fig. 4. Relationship between overlap ratio with PASA assembly and the number of genes. A substantial fraction of predicted genes has little overlap ratio with PASA assembly transcripts. By selecting genes of ≥ 5% overlap with PASA assembly, we remove putative false-positive genes while retaining genes with expression evidence. [file 12864_2024_10929_MOESM5_ESM.docx]

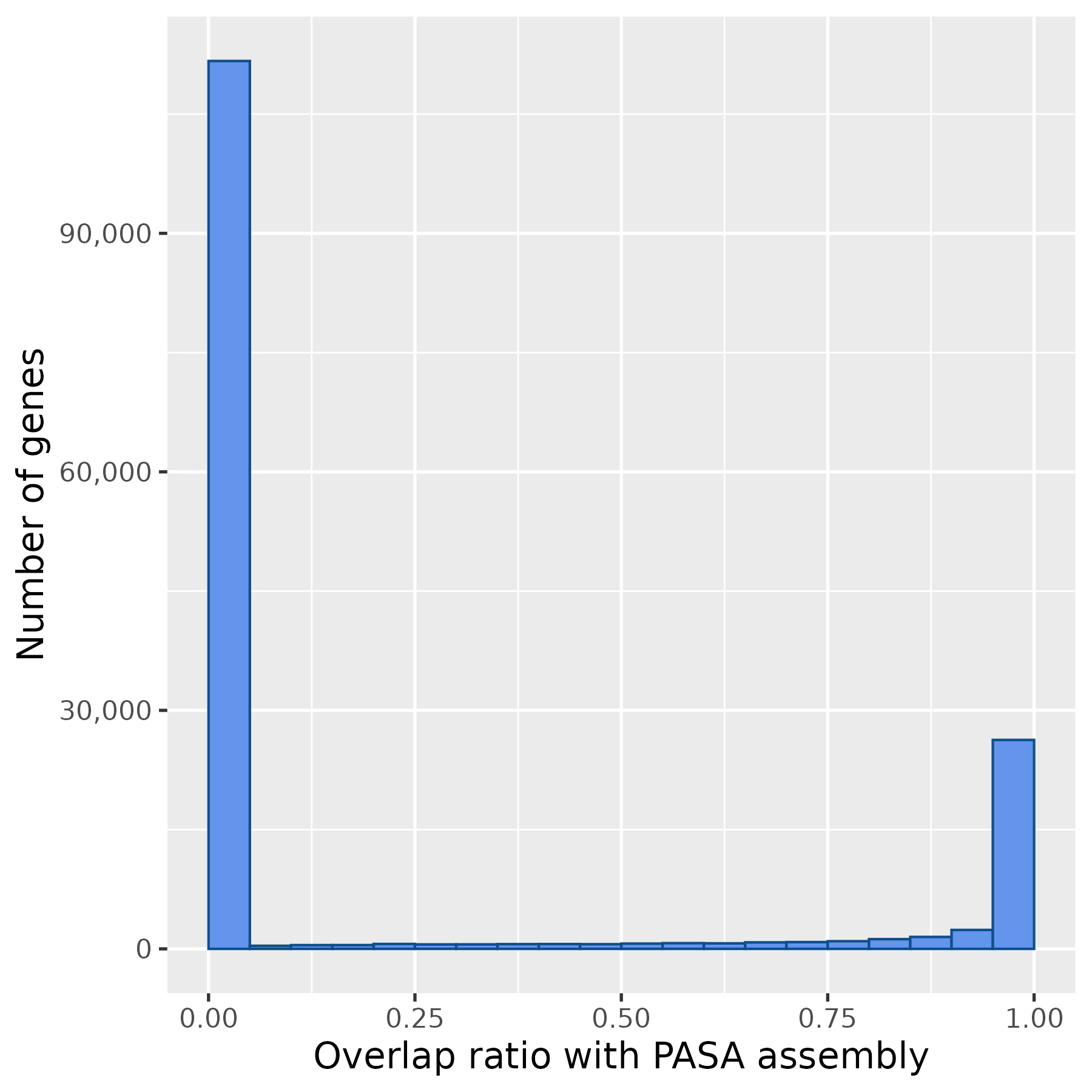


**Supplementary Figure 4** Relationship between overlap ratio with PASA assembly and the number of genes.

A substantial fraction of predicted genes has little overlap ratio with PASA assembly transcripts. By selecting genes of ≥5% overlap with PASA assembly, we remove putative false-positive genes while retaining genes with expression evidence.
