# Supplementary material for: A chromosome-level genome assembly of a model conifer plant, the Japanese cedar, Cryptomeria japonica D. Don
Source: BMC Genomics. 2024 Nov 5;25:1039. doi: 10.1186/s12864-024-10929-4 (PMC11539532; doi:10.1186/s12864-024-10929-4)
Supplement: Supplementary file 9 — Supplementary Material 9: Fig. 5. Pulsed-field gel electrophoresis (PFGE) of high molecular weight DNA from Cryptomeria japonica flushing buds. Size distribution of high molecular weight (HMW) DNA from Cryptomeria japonica samples (labeled 1727 and 1728) was assessed using PFGE. HMW DNA samples ranging from 20 to 200 ng were electrophoresed through a 1% agarose gel over a 15 h period using the Bio-Rad CHEF Mapper system. A lambda ladder and a 5-kbp ladder (labeled as λ and 5 kb, respectively) were employed as molecular size standards that flanked the lanes containing the HMW DNA samples. Orange lines corresponding to the bands of the size standards (in kbp). [file 12864_2024_10929_MOESM9_ESM.docx]

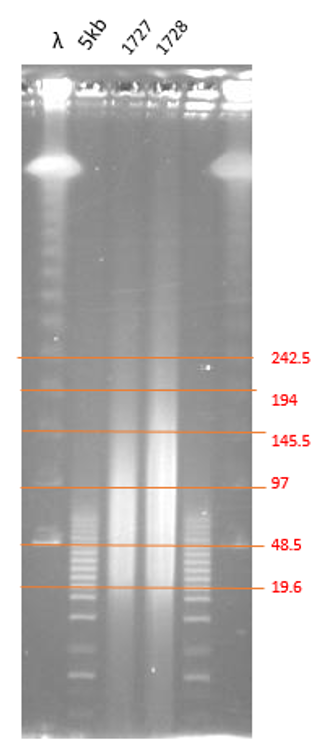


**Supplementary Figure 5** Pulsed-field gel electrophoresis (PFGE) of high molecular weight DNA from *Cryptomeria japonica* flushing buds.

Size distribution of high molecular weight (HMW) DNA from *Cryptomeria japonica* samples (labeled 1727 and 1728) was assessed using PFGE. HMW DNA samples ranging from 20–200 ng of were electrophoresed through a 1% agarose gel over a 15 hour period using the Bio-Rad CHEF Mapper system. A lambda ladder and a 5-kbp ladder (labeled as λ and 5kb, respectively) were employed as molecular size standards that flanked the lanes containing the HMW DNA samples. Orange lines corresponding to the bands of the size standards (in kbp).
