# Supplementary material for: A chromosome-level genome assembly of a model conifer plant, the Japanese cedar, Cryptomeria japonica D. Don
Source: BMC Genomics. 2024 Nov 5;25:1039. doi: 10.1186/s12864-024-10929-4 (PMC11539532; doi:10.1186/s12864-024-10929-4)
Supplement: Supplementary file 13 — Supplementary Material 13: Fig. 6. Base composition report of the HiFi reads using fastp (before trimming). The x-axis represents the position in the read ranging from positions 1 to 50,000, and y-axis shows the base content ratios for each nucleotide (A, T, C, and G, represented by pastel yellow, purple, light green, and blue, respectively). The N (red) and GC (black) percentages are shown. The relatively horizontal lines across the read positions after the 20th base for A, T, C, and G bases indicate the absence of significant base composition bias. In contrast, at the beginning of the read up to 20th base may be biased due to artificial sequences. [file 12864_2024_10929_MOESM13_ESM.docx]

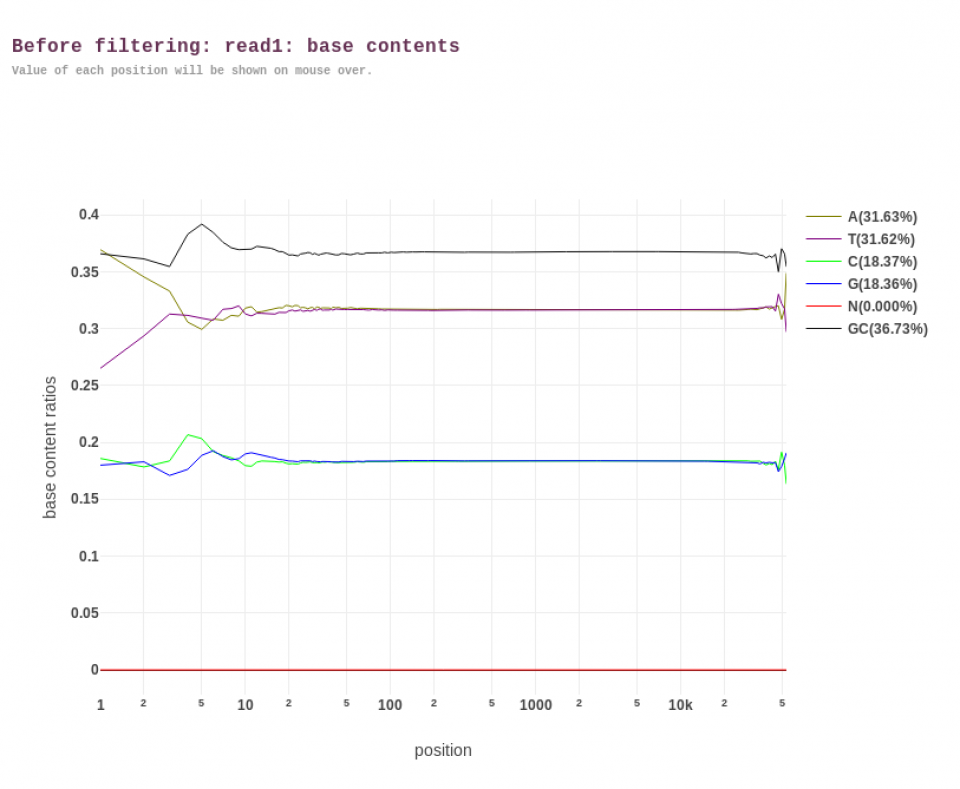


**Supplementary Figure 6** Base composition report of the HiFi reads using fastp (before trimming).

The x-axis represents the position in the read ranging from positions 1 to 50,000, and y-axis shows the base content ratios for each nucleotide (A, T, C, and G, represented by pastel yellow, purple, light green, and blue, respectively). The N (red) and GC (black) percentages are shown. The relatively horizontal lines across the read positions after the 20th base for A, T, C, and G bases indicate the absence of significant base composition bias. In contrast, at the beginning of the read up to 20th base may be biased due to artificial sequences.
