# Supplementary material for: A chromosome-level genome assembly of a model conifer plant, the Japanese cedar, Cryptomeria japonica D. Don
Source: BMC Genomics. 2024 Nov 5;25:1039. doi: 10.1186/s12864-024-10929-4 (PMC11539532; doi:10.1186/s12864-024-10929-4)
Supplement: Supplementary file 14 — Supplementary Material 14: Fig. 7. Base composition report of the HiFi reads using fastp (after trimming). The x-axis represents the position in the read ranging from 1 to 50,000, and the y-axis shows the base content ratios for each nucleotide (A, T, C, and G, represented by pastel yellow, purple, light green, and blue, respectively). The N (red) and GC (black) percentages are shown. The relatively horizontal lines across the overall read positions suggest that no significant bias in base composition after trimming. [file 12864_2024_10929_MOESM14_ESM.docx]

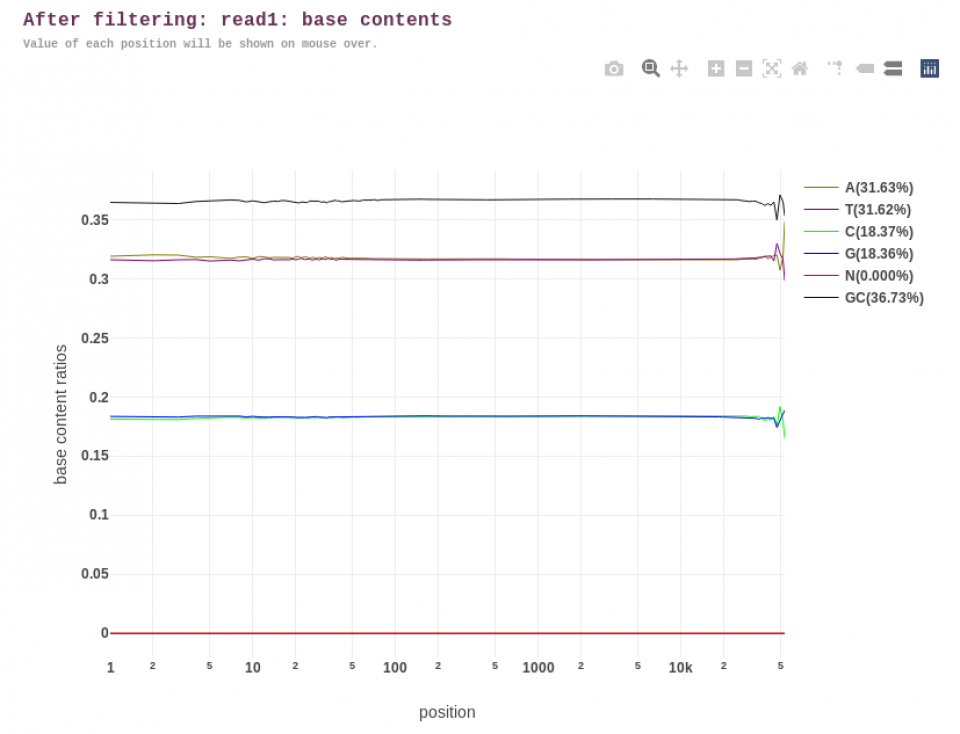


**Supplementary Figure 7** Base composition report of the HiFi reads using fastp (after trimming).

The x-axis represents the position in the read ranging from 1 to 50,000, and the y-axis shows the base content ratios for each nucleotide (A, T, C, and G, represented by pastel yellow, purple, light green, and blue, respectively). The N (red) and GC (black) percentages are shown. The relatively horizontal lines across the overall read positions suggest that no significant bias in base composition after trimming.
